# Micro-scale, mid-scale, and macro-scale in global seismicity identified by empirical mode decomposition and their multifractal characteristics

**DOI:** 10.1038/s41598-018-27567-y

**Published:** 2018-06-15

**Authors:** Nicholas V. Sarlis, Efthimios S. Skordas, Apostolis Mintzelas, Konstantina A. Papadopoulou

**Affiliations:** 1 Section of Solid State Physics, Department of Physics, National and Kapodistrian University of Athens, Panepistimiopolis, Zografos, 157 84 Athens Greece; 2 Solid Earth Physics Institute, Department of Physics, National and Kapodistrian University of Athens, Panepistimiopolis, Zografos, 157 84 Athens Greece

## Abstract

The magnitude time-series of the global seismicity is analyzed by the empirical mode decomposition giving rise to 14 intrinsic mode functions (IMF) and a trend. Using Hurst analysis one can identify three different sums of these IMFs and the trend which exhibit distinct multifractal behaviour and correspond to micro-, mid- and macro-scales. Their multifractal detrended fluctuation analysis reveals that the micro-scale time-series exhibits anticorrelated behaviour in contrast to the mid-scale one which is long-range correlated. Concerning the mid-scale one, in the range of 30 to 300 consecutive events the maximum entropy method power spectra indicates that it exhibits an 1/*f*^*α*^ behaviour with *α* close to 1/3 which is compatible with the long-range correlations identified by detrended fluctuation analysis during periods of stationary seismicity. The results have been also verified to hold regionally for the earthquakes in Japan and shed light on the significance of the mid-scale of 30 to 300 events in the natural time analysis of global (and regional) seismicity. It is shown that when using the mid-scale time-series only, we can obtain results similar to those obtained by the natural time analysis of global seismicity when focusing on the prediction of earthquakes with *M* ≥ 8.4.

## Introduction

Earthquake (EQ) is a common physical phenomenon that is related with the tectonic structure of the solid Earth crust on which we live and whose prediction^1^ is very important for human welfare. Seismicity exhibits complexity in many aspects giving rise to correlations between hypocenters, occurrence times, and EQ magnitudes *M* which have been the subject of several studies^2–28^. Here, we focus on the analysis of EQ magnitude time-series for which we have shown^13,14^ that there exist correlations between successive EQs of magnitude 7.0 or larger in a global scale. In an independent study, Fan and Lin^27^ analysed the EQ magnitude time-series in Southern California by the empirical mode decomposition (EMD)^29–33^ method and identified the presence of three different time scales: The micro-scale, the mid-scale and the macro-scale. The identification of these three time scales has been based on the different multifractal behaviour observed through multifractal detrended fluctuation analysis (MFDFA)^34^ for the corresponding time-series. Consequently, the following important question arises: If in general the EQ magnitude time-series is a result of the superposition of these three distinct time-series of different spectral content (micro-, mid- and macro-scale), it is of major importance to investigate here which of these three scales is of primary usefulness for EQ prediction. In particular, we shall show that the mid-scale is the most appropriate scale to achieve such a purpose by analyzing the seismicity in a new time domain termed natural time^35–39^.

Natural time analysis (NTA) has been introduced^35^ almost fifteen years ago and enables the identification of novel dynamical features hidden behind the time-series resulting from complex systems^37^. Taking the view that EQs are critical phenomena, the quantity by which one can identify the approach of a dynamical system to the state of criticality is termed order parameter. We recall that according to the definition of this parameter (see p. 449 of ref.^40^): when a body passes through the phase transition point, we can define a quantity called the order parameter, in such a way that it takes non-zero (positive or negative) values in the unsymmetrical phase and is zero in the symmetrical phase. For the case of seismicity, NTA enabled^41^ the introduction of an order parameter labeled *κ*_1_. This order parameter abruptly changes to zero upon the occurrence of a strong EQ (which corresponds to the new phase, the “disordered” phase) while it remains non-zero when no such strong EQ occurs (“ordered” phase) (see also pp. 249–254 of ref.^37^). Later studies of this order parameter have shown^18,42–44^ that its fluctuations *β*_*W*_(*κ*_1_) when studied within excerpts of the EQ catalog comprising *W* consecutive EQs, they exhibit characteristic minima that almost coincide^17,45,46^ with the observation of Seismic Electric Signals (SES) activities^47,48^ which are series of low-frequency (≤1 Hz) variations of the electric field of the Earth that appear a few weeks up to six months before EQs in Greece^48–53^ and Japan^36,54–57^. Interestingly, the average lead time of SES activities corresponds to the average time for the observation of the precursory minima of *β*_*W*_(*κ*_1_), thus showing the existence of a characteristic time scale of the order of a few months that dominates the last stage of the preparation of a strong EQ. In addition, minima of *β*_*W*_(*κ*_1_) have been identified^23^ in global seismicity by selecting *W* values corresponding to the average number of EQs that occur within a few months which are precursory to EQs of magnitude class 9.

The present work is structured as follows: we first examine the existence of the micro-, mid- and macro-scale in global seismicity by using the rescaled range (R/S) or Hurst^58^ analysis together with EMD and MFDFA. Once this has been established, we also show that the same analysis can be applied to the case of EQs in Japan for which an EQ prediction scheme, similar to the aforementioned one for global seismicity, has been proposed^18,24^. The examination of the spectral content of the micro-, mid- and macro-scale time-series reveals that the mid-scale exhibits a behaviour compatible with that found^8,59,60^ during the regimes of stationary seismic activity when focusing in the range of 30 to 300 consecutive events. This constitutes the first indication that the mid-scale might be responsible for the aforementioned precursory changes in the fluctuations of the order parameter of seismicity since the related *W* values also lie within this range. Finally, to verify this connection we propose an EQ prediction scheme that uses only the mid-scale time-series for the prediction of strong EQs in global scale.

## Results

**Figure 1:**
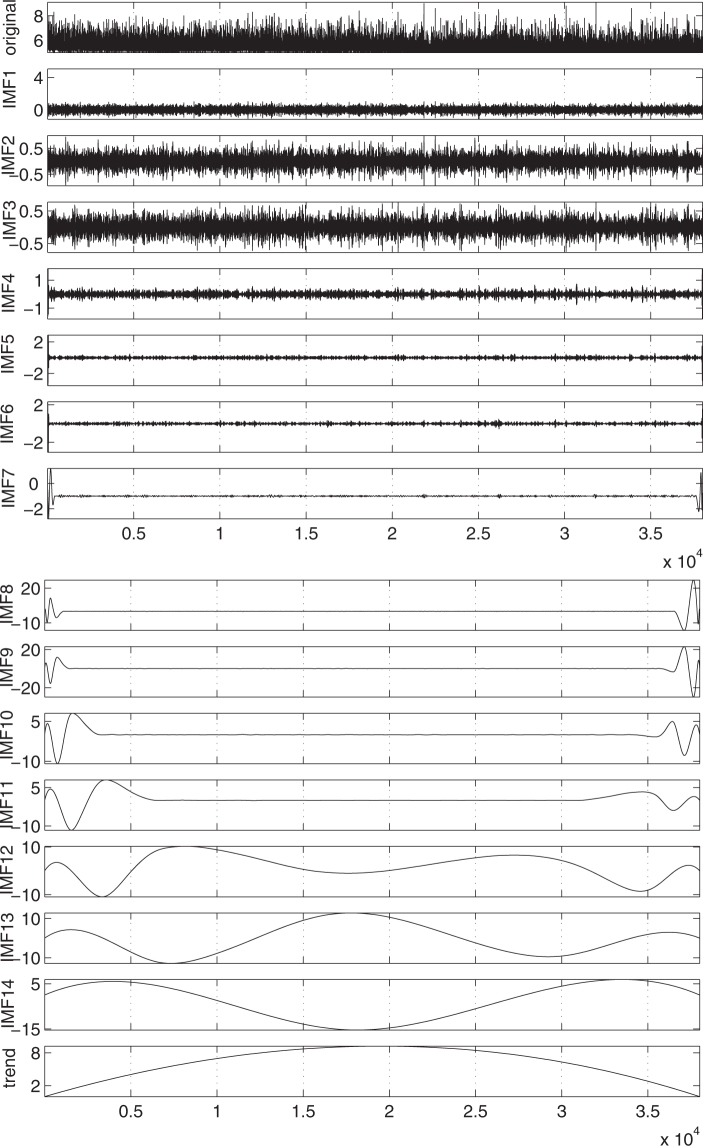
EMD of the magnitude time-series of GCMT in 14 IMFs and a trend. The data correspond to the period from 1 January 1976 to 1 October 2014.

**Figure 2:**
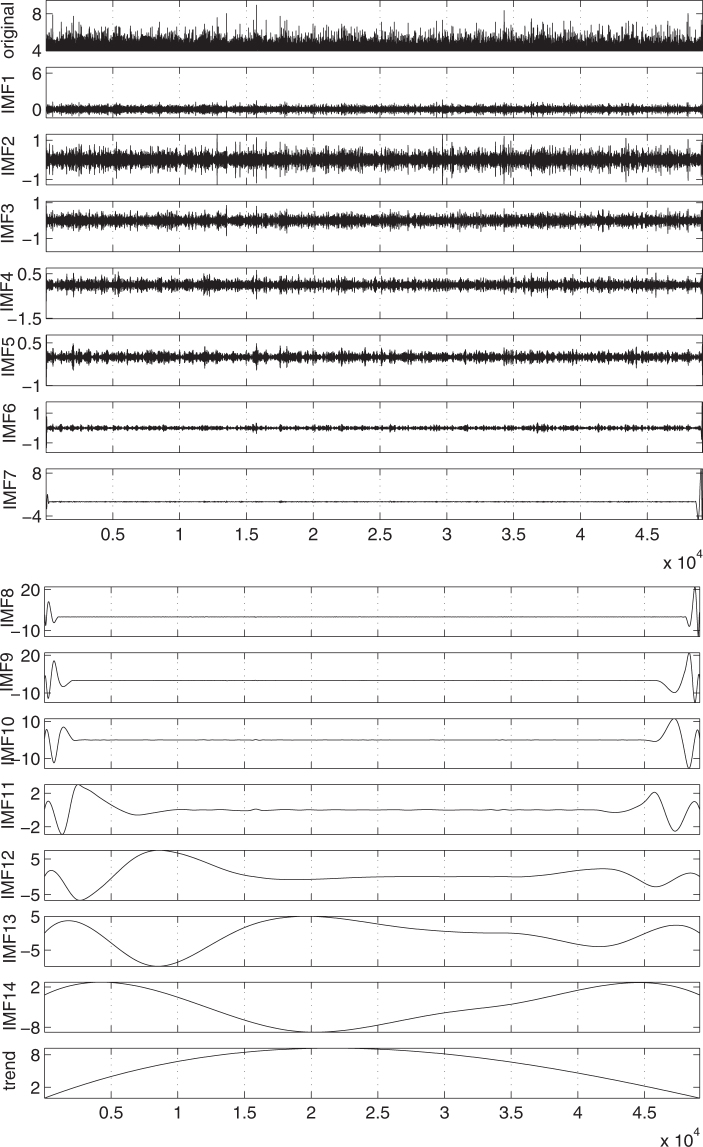
EMD of the magnitude time-series of Japan in 14 IMFs and a trend.

As mentioned in the Introduction, here we focus on the EQ magnitude time-series. The data describing global seismicity come from the Global Centroid Moment Tensor Project^61,62^ and the corresponding EQ magnitude time-series, called for brevity GCMT, is shown in the upper panel of Fig. 1. The latter time-series is the one obtained for *M* ≥ 5.0 (for further details see Methods). Similarly, the EQ magnitude time-series used for the regional study of Japan is the one used in refs^18,20,24^ and is shown in the upper panel of Fig. 2 (for details see Methods).

### Identification of the micro-, mid- and macro-scale

In order to identify the micro-, mid- and macro-scale components in the EQ magnitude time-series we first employ EMD (see Methods). EMD is a method that separates a time-series into various component time-series called Intrinsic Mode Functions (IMF) and a trend. IMFs satisfy appropriate criteria so that their Hilbert transform may give rise^29^ to a well defined *instantaneous* amplitude and frequency. In general, they are oscillating functions but not of a given frequency as in the case of Fourier transform or of a given shape as in the case of the wavelet transform. Even in the case of white noise, the total number of IMFs of a data set is close to log_2_ *N* with *N* the number of total data points^63^. Figure 1 depicts the EMD of GCMT into 14 IMFs together with the trend. Similar results are obtained for Japan as shown in Fig. 2.

**Figure 3:**
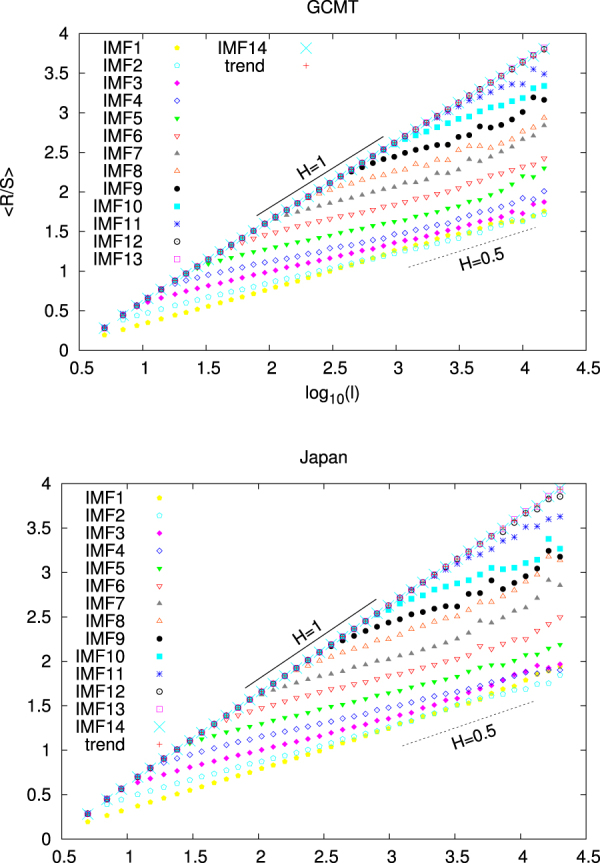
Hurst analysis for the magnitude time-series of GCMT and Japan. The solid and the dashed line correspond to *H* = 1 and *H* = 0.5, respectively.

**Figure 4:**
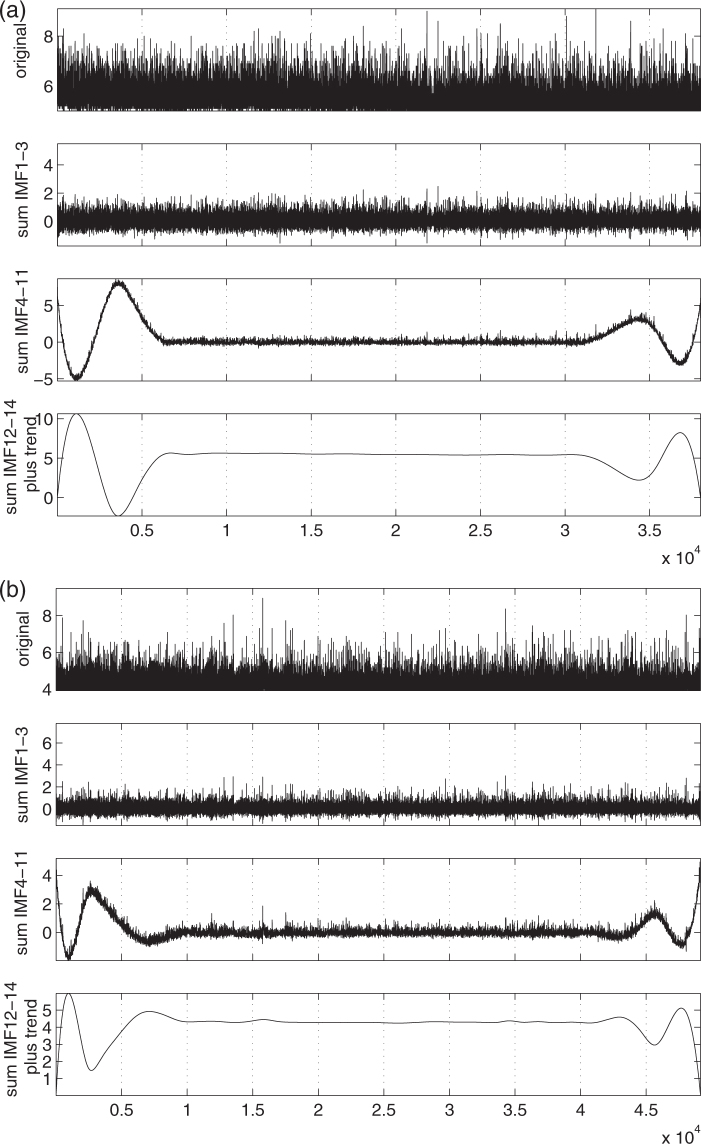
Separation of magnitude time-series into three components. The magnitude time-series of GCMT (**a**) and Japan (**b**) and their decomposition into micro-, mid- and macro-scale time-series.

A basic tool for the identification of long-range dependence in time-series is the R/S or Hurst^58^ analysis (see Methods). This examines how the ratio of the range over the standard deviation of the profile of a time-series varies with the scale *l*. If R/S is a power law of *l*, the corresponding exponent is called Hurst exponent *H*. The time-series exhibits long-range correlation when *H* is larger than 0.5 whereas *H* < 0.5 points to anticorrelation. The R/S analysis for the IMFs and the trend of the GCMT is shown in the upper panel of Fig. 3. We observe that for the IMFs 12, 13, 14 and the trend a straight line of unit slope results in the log-log plot of R/S versus *l*. On the other side, IMFs 1, 2, and 3 show again a straight line behaviour but with slopes close to 0.5 or even lower. The latter behaviour can be identified^27^ as indicative of the IMFs that constitute the micro-scale of the EQ magnitude time-series. The R/S analysis of the IMFs 4 to 11 clearly indicates the existence of a cross-over in which the slope in the log-log diagram abruptly changes from *H* = 1 to values that may be even smaller than *H* = 0.5. Following ref.^27^, this dual fractal behaviour shows that these IMFs constitute the mid-scale time-series. Figure 4(a) reveals how the EQ magnitude time-series of GCMT can be decomposed into three component time-series the micro-scale (the sum of IMF1 to IMF3), the mid-scale (the sum of IMF4 to IMF11) and the macro-scale (the sum of IMF12 to IMF14 plus the trend) time-series. A similar analysis for Japan is also shown in Fig. 4(b).

**Figure 5:**
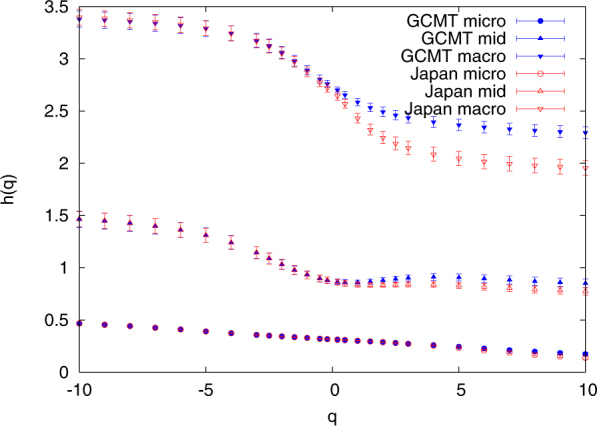
The generalized Hurst exponents *h*(*q*) versus *q*. They result from MFDFA by fitting in the range *l* = 10–10^3^ for the micro- and mid-scales whereas *l* = 300–10^4^ for the macro-scale. The results for GCMT and Japan are depicted by the (blue) closed and the (red) open symbols with errorbars, respectively.

**Figure 6:**
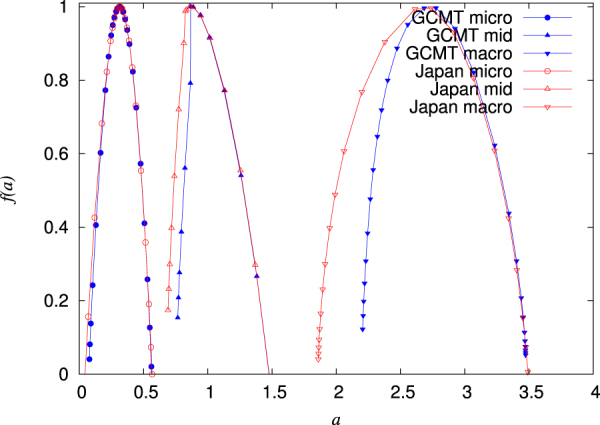
The singularity spectra *f*(*a*) versus *a*. They come from the MFDFA *h*(*q*) presented in Fig. 5. The results for GCMT and Japan are depicted by the (blue) closed and the (red) open symbols, respectively.

Comparing these results for GCMT and Japan, we note that apart from a similar behaviour in the R/S analysis, the micro-, mid- and macro-scale time-series exhibit^27^ distinct multifractal behaviours, see for example Fig. 8 of ref.^27^. The multifractal behaviour of these three time-series has been studied by MFDFA (for more details see Methods) and the generalized Hurst exponent *h*(*q*) is shown in Fig. 5. We observe that for both the global and the Japanese seismicity the micro-, mid- and macro-scale time-series exhibit a similar behaviour which can be also seen in Fig. 6 where we depict the singularity spectrum *f*(*a*). Figures 5 and 6 undoubedtly reveal that the micro-scale exhibits an anticorrelated behaviour similar to that found by Fan and Lin^27^ for Southern California, while the mid-scale time-series is long-range correlated. Such a similarity in the multifractal properties does not necessarily imply that the details of the dynamics of global seismicity are the same as that of Japan (obviously Japanese seismicity is dominated by the Pacific plate subduction zone, and is simply a part of the global seismicity). Such details are actually present (e.g., for the mid-scale time-series see Fig. S1).

### Spectral study of the micro-, mid- and macro-scale

**Figure 7:**
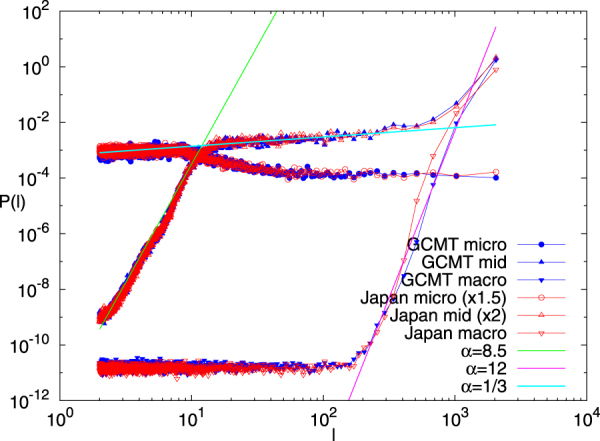
The MEM spectra for the micro-, mid- and macro-scale. The results for GCMT and Japan are depicted by the (blue) closed and the (red) open symbols, respectively. The results of Japan for micro and mid-scale have been multiplied by 1.5 and 2, respectively. The lines have been drawn as a guide to the eye. We can see that the results for the mid-scale exhibit an 1/*f*^*α*^ behaviour with *α* ≈ 1/3 in the range of scales from 30 to 300.

Figure 7 depicts the Fourier power spectral density *P*(*l*) of the micro-, mid- and macro-scale time-series, estimated by the Maximum Entropy Method (MEM)^64^ as implemented by the TISEAN package^65^, versus the scale *l* which now stands for the period of the Fourier transform. We observe that the frequency content of each of the three scales falls within well defined margins which for the mid-scale, for example, lie between *l* = 10 and *l* = 400 events. Especially, in the range of 30 to 300 consecutive events the mid-scale time-series power spectrum exhibits an 1/*f*^*α*^ behaviour with *α* close to 1/3. If we now recall that *α* is related to the detrended fluctuation analysis exponent^66,67^ *a*_*DFA*_ by means of the relation^68^ *α* = 2*a*_*DFA*_ − 1, we obtain *a*_*DFA*_ ≈ 2/3. Since 0.5 < *α*_*DFA*_ < 1 this value reflects that there exist long range temporal correlations between EQ magnitudes^11^ and it is very close to the corresponding value found^8,11,59,60^ during the regimes of stationary seismic activity.

### The significance of the mid-scale

Since the mid-scale exhibits, as mentioned, a long-range correlated behaviour and spectral characteristics similar to those found in the regimes of stationary seismic activitiy, could it lie behind the precursory minima of the fluctuations of the order parameter of seismicity that have been observed for EQ catalog excerpts corresponding to the aforementioned characteristic time scale of a few months consisting of *W* = 100 and *W* = 160 consecutive EQs?

**Figure 8:**
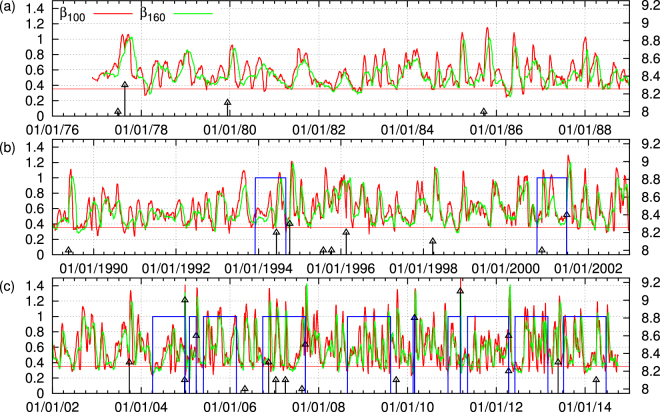
Natural time analysis of global seismicity. The variabilities (left scale) *β*_100_ (red) and *β*_160_ (green) versus conventional time for the periods: (**a**) 1 January 1976 to 31 December 1988, (**b**) 1 January 1989 to 31 December 2002, and (**c**) 1 January 2002 to 1 October 2014. The thin blue line corresponds to the alarm (1 = on and 0 = o, left scale) lasting nine months after the occurrence of min(*β*_160_) when using (*β*_0_, *r*_1_, *r*_2_) = (0.353, 1.060, 1.135) for the prediction^23^ of the occurrence time of EQs with *M* ≥ 8.4 which are shown with the vertical lines ending at black triangles (right scale). The percentange of the total alarm time is *τ* = 21%. The horizontal red line corresponds to *β*_0_ = 0.353.

Let us recall that NTA allows the introduction of an order parameter for seismicity labeled *κ*_1_, as mentioned. The value of *κ*_1_ depends on the way the total energy is emitted during *N* EQs (see Methods) and once we have an EQ catalog excerpt comprising of *W* EQs, we can estimate an ensemble of *κ*_1_-values. Then, the fluctuations of *κ*_1_ can be quantified by the ratio^60^ *β*_*W*_(*κ*_1_) ≡ *σ*_*W*_(*κ*_1_)/*μ*_*W*_(*κ*_1_) where *μ*_*W*_(*κ*_1_) and *σ*_*W*_(*κ*_1_) correspond to the average value and the standard deviation of the distribution of *κ*_1_ within this ensemble, respectively. By sliding the window of *W* EQs through the whole EQ catalog we can obtain a picture of how the order parameter of seismicity fluctuations change with time, e.g. see Fig. 8. If the window length *W* is selected so that to correspond to the average lead time of SES, it has been shown^18,23,42–44^ that minima of the aforementioned fluctuations are observed before the stronger EQs. For the case of global seismicity which is the subject of our study, it was found^23^ that such minima can be uniquely identified by studying the local minima of *β*_100_ and *β*_160_. A local minimum of either *β*_100_ or *β*_160_ is one that remains a minimum for at least its 15 previous and 15 future values (cf. in view of the average rate of 80 EQs/month, this corresponds to a time period of almost two weeks, see Methods). For a precursory variability minimum to be observed, *β*_100_ and *β*_160_ should exhibit their minima simultaneously. Following ref.^20^, we require at least 90% of the EQs that are included in the calculation of the local *β*_100_ minimum are also included in the calculation of the local *β*_160_ minimum. Once simultaneous local *β*_100_ and *β*_160_ minima are observed, we examine whether their ratio *r* ≡ min(*β*_160_)/min(*β*_100_) lies within the margins defined by the precursory to strong EQs variability minima. This is understood in the context that, since EQs are considered critical phenomena as mentioned in the Introduction, the origin of *β*_*W*_ minima in these cases stems from similar criticality thus having the same dependence on the scale *W*. A selection of the margins (*r*_1_=)1.060 < *r* < 1.135 (=*r*_2_) allows the prediction of all *M* ≥ 8.4 EQs as mentioned in the first paragraph of the Appendix of ref.^23^. In order to minimize the number of false alarms, one also imposes a threshold *β*_0_ in the minima of *β*_100_ to be examined by the aforementioned procedure. Table 1 shows all the 19 minima found by this procedure when selecting min(*β*_100_) below (*β*_0_=)0.353, which is the shallowest minimum *β*_100_ value observed before an *M* ≥ 8.4 EQ. We observe that all 7 EQs with *M* ≥ 8.4 are preceded within nine months by variability minima, while 11 more (false alarm) cases correspond to EQs with *M* ≥ 7.6. Figure 8 depicts the results of this analysis together with the alarm which is set to 1 (on) after a precursory min(*β*_160_) has been observed and lasts either 9 months or up to the strong EQ occurrence if the observed magnitude satisfies *M* ≥ 8.4. A calculation of the total alarm time shows that it corresponds to (*τ*≡)21% of the total time studied (13,734 days).

**Table 1:** The EQs that are preceded within 9 months from the 19 variability minima identified when studying the global seismicity using the parameters (*β*_0_, *r*_1_, *r*_2_) = (0.353, 1.060, 1.135) for the prediction^23^ of the occurrence time of all EQs with *M* ≥ 8.4.

EQ date	Lat. (°N)	Long. (°E)	*M*	min(*β*_100_)	min(*β*_160_)	*r*	Δ*t*_160_ (months)
19940609	−13.83	−67.56	8.2	0.351 (19931027)	0.383 (19931203)	1.092	7.5
20010623	−16.26	−73.64	8.4	0.352 (20001006)	0.382 (20001004)	1.086	8.7
20041226	3.30	95.78	9.0	0.227 (20040405)	0.243 (20040405)	1.071	8.8
20050328	2.09	97.11	8.6	0.161 (20050128)	0.170 (20050202)	1.060	2.0
20050613	−19.99	−69.20	7.8	0.337 (20050517)	0.357 (20050528)	1.060	0.9
20061115	46.57	153.29	8.3	0.351 (20060928)	0.381 (20060928)	1.086	1.6
”	”	”	8.3	0.342 (20061001)	0.369 (20061015)	1.078	1.5
20070912	−4.44	101.37	8.5	0.277 (20061202)	0.297 (20061220)	1.073	9.5
20090103	−0.41	132.88	7.7	0.280 (20080825)	0.305 (20080825)	1.088	4.4
20090715	−45.76	166.56	7.8	0.342 (20081116)	0.377 (20081116)	1.101	8.0
20100227	−35.85	−72.71	8.8	0.232 (20100201)	0.246 (20100216)	1.063	0.9
20110311	38.32	142.37	9.1	0.237 (20101129)	0.264 (20101130)	1.114	3.4
”	”	”	9.1	0.347 (20110306)	0.389 (20110306)	1.122	0.2
20110706	−29.54	−176.34	7.6	0.347 (20110510)	0.380 (20110510)	1.096	1.9
”	”	”	7.6	0.288 (20110605)	0.305 (20110618)	1.062	1.0
20120411	2.33	93.06	8.6	0.285 (20110727)	0.323 (20110804)	1.134	8.6
20130206	−10.80	165.11	7.9	0.279 (20120520)	0.305 (20120603)	1.095	8.7
20140401	−19.61	−70.77	8.1	0.346 (20130619)	0.389 (20130708)	1.124	9.5
20140401	−19.61	−70.77	8.1	0.348 (20130924)	0.380 (20130924)	1.092	6.3

Δ*t*_160_ corresponds to the time period that elapsed from the observation of min(*β*_160_) and the EQ occurrence and is measured in months. The dates of EQs as well as the dates of minima appearance are shown in the format YYYYMMDD.

**Figure 9:**
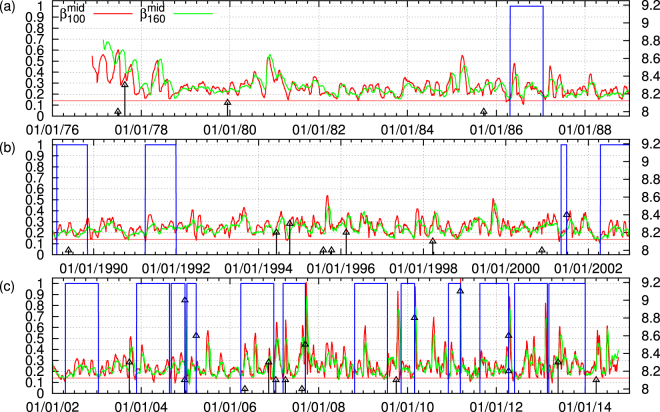
Analysis of the mid-scale time-series of global seismicity in natural time. The variabilities (left scale) ${\beta }_{100}^{mid}$ (red) and ${\beta }_{160}^{mid}$ (green) versus conventional time for the periods: (**a**) 1 January 1976 to 31 December 1988, (**b**) 1 January 1989 to 31 December 2002, and (**c**) 1 January 2002 to 1 October 2014. The thin blue line corresponds to the alarm (1 = on and 0 = off, left scale) lasting nine months after the occurrence of ${\rm{\min }}({\beta }_{160}^{mid})$ when using (*β*_0_, *r*_1_, *r*_2_) = (0.140, 1.13, 1.54) for the prediction of the occurrence time of EQs with *M* ≥ 8.4 which are shown with the vertical lines ending at black triangles (right scale). The percentange of the total alarm time is *τ* = 24%. The horizontal red line corresponds to *β*_0_ = 0.140.

In order to answer the aforementioned question, i.e., whether the study of the mid-scale alone enables the detection of the precursory minima of the order parameter fluctuations, we analyzed in natural time only the mid-scale time-series of global seismicity and constructed ${\beta }_{100}^{mid}$ and ${\beta }_{160}^{mid}$ which are shown in Fig. 9. A behaviour similar to that observed in Fig. 8 emerges, although the values of ${\beta }_{100}^{mid}$ and ${\beta }_{160}^{mid}$ are usually two to three times smaller than those of *β*_100_ and *β*_160_. We further investigated the possibility to predict the *M* ≥ 8.4 strong EQs by using only ${\beta }_{100}^{mid}$ and ${\beta }_{160}^{mid}$ when employing (*β*_0_, *r*_1_, *r*_2_) = (0.140, 1.13, 1.54). We obtained the 17 precursory minima shown in Table 2. These minima correspond to all the 7 EQs with *M* ≥ 8.4 while there are 10 additional minima which are precursory to EQs with *M* ≥ 7.3. The percentange of total alarm time is now *τ* = 24% in comparison with the previous value of 21%. Thus, we observe that even though EMD has removed a lot of variation from the original magnitude time-series, the valuable information for EQ prediction still remains in the mid-scale time-series pointing to the importance of natural time-scales of 30 to 300 consecutive EQs that correspond to the mean value of the SES lead time. One may argue that the edge effects that give rise to the oscillatory modes at the end and at the begining of mid-scale time-series of global seismicity (see Fig. 4(a)) may affect the above result of *τ* = 24%. In order to answer this question, Figs S2, S3, S4, S5, S6, S7, S8 and S9 show the results obtained by various techniques, including the Ensemble EMD (EEMD)^63^, that aim to the elimination of edge effects, e.g., see the third panel in Figs S4 and S6(b). All these results lead to *τ* values in the range 24% to 27% (see Figs S5, S7 and S9), which are close to the value *τ* = 21% found for the original time-series and in addition are markedly smaller than the value of *τ* = 36% obtained when studying ${\beta }_{100}^{micro}$ and ${\beta }_{160}^{micro}$ and analyzing the micro-scale time-series in natural time.

**Table 2:** The EQs that are preceded within 9 months from the 17 variability minima identified when studying the mid-scale time-series of the global seismicity using the parameters (*β*_0_, *r*_1_, *r*_2_) = (0.140, 1.13, 1.54) for the prediction of the occurrence time of all EQs with *M* ≥ 8.4.

EQ date	Lat. (°N)	Long. (°E)	*M*	min(${{\boldsymbol{\beta }}}_{{\bf{100}}}^{{\boldsymbol{mid}}}$)	min(${{\boldsymbol{\beta }}}_{{\bf{160}}}^{{\boldsymbol{mid}}}$)	*r*	Δ*t*_160_ (months)
19860507	51.41	−174.83	7.9	0.130 (19860323)	0.151 (19860425)	1.161	1.5
19890523	−52.24	160.20	8.0	0.130 (19890209)	0.153 (19890209)	1.175	3.4
19911222	45.47	151.05	7.6	0.119 (19910405)	0.160 (19910404)	1.336	8.7
20010623	−16.26	−73.64	8.4	0.117 (20010419)	0.160 (20010505)	1.366	2.2
20021103	63.52	−147.44	7.8	0.110 (20020413)	0.135 (20020418)	1.231	6.8
20031227	−22.01	169.77	7.3	0.123 (20031112)	0.180 (20031124)	1.458	1.5
20041226	3.30	95.78	9.0	0.134 (20040830)	0.205 (20040904)	1.532	3.9
20050328	2.09	97.11	8.6	0.132 (20050101)	0.170 (20050111)	1.295	2.9
20061115	46.57	153.29	8.3	0.126 (20060331)	0.145 (20060331)	1.154	7.6
20070912	−4.44	101.37	8.5	0.140 (20070312)	0.185 (20070314)	1.319	6.1
20090715	−45.76	166.56	7.8	0.134 (20081006)	0.177 (20081022)	1.321	9.4
20100227	−35.85	−72.71	8.8	0.122 (20091026)	0.162 (20091109)	1.325	4.1
20110311	38.32	142.37	9.1	0.127 (20101130)	0.144 (20101203)	1.133	3.4
20120411	2.33	93.06	8.6	0.128 (20110820)	0.179 (20110820)	1.402	7.8
20130206	−10.80	165.11	7.9	0.112 (20120519)	0.147 (20120530)	1.311	8.8
20130524	54.89	153.22	8.3	0.129 (20130225)	0.191 (20130309)	1.483	2.9
”	”	”	8.3	0.092 (20130405)	0.139 (20130406)	1.522	1.6

Δ*t*_160_ corresponds to the time period that elapsed from the observation of ${\rm{\min }}({\beta }_{160}^{mid})$ and the EQ occurrence and is measured in months. The dates of EQs as well as the dates of minima appearance are shown in the format YYYYMMDD.

## Discussion

**Figure 10:**
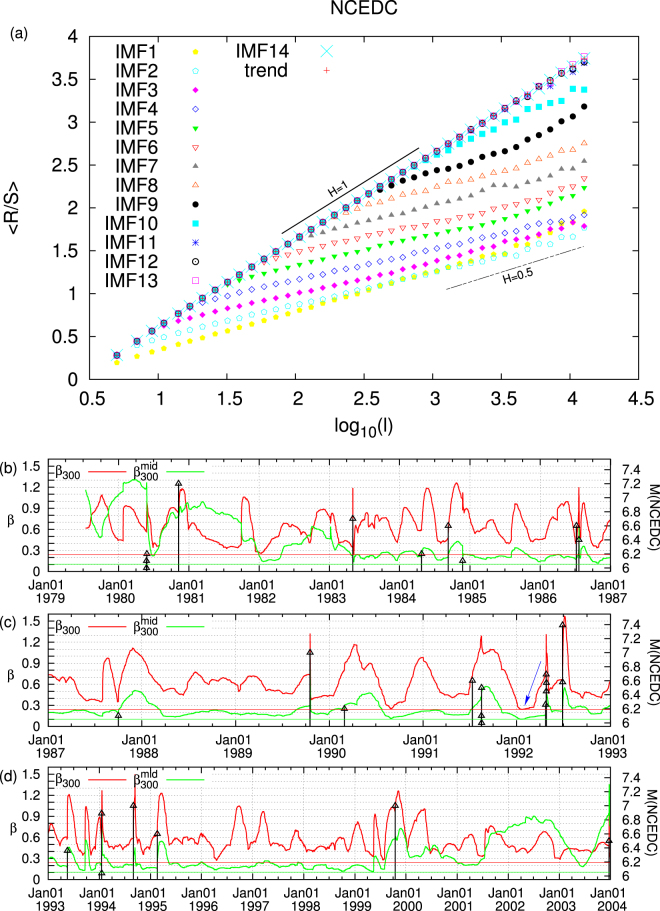
Results from the analysis of the NCEDC magnitude time-series. Panel (a) shows the Hurst analysis of the 14 IMFs and the trend while the lower three panels depict *β*_300_ (red) and ${\beta }_{300}^{mid}$ (green) versus conventional time. The EQs with *M* ≥ 6 are shown with the vertical lines ending at black triangles (right scale). The blue arrow indicates the global minimum observed before Landers EQ. The horizontal red and green lines correspond to the minimum values of *β*_300_ and ${\beta }_{300}^{mid}$.

We have seen that the magnitude time-series of the global seismicity (as well as that of Japan) can be decomposed into three time-series which can be identified as sums of the IMFs and the trend obtained by EMD (see Figs 1 and 2). It is the Hurst analysis (see Fig. 3) of the IMFs that determines which IMF belongs to each of the three time-series in agreement with the corresponding analysis^27^ for California. The general rule is that the lower order IMFs exhibit an almost antipersistent behaviour resulting to a single Hurst exponent which is smaller than 0.5. Their sum constitutes the micro-scale time-series. On the other hand, the higher IMFs together with the trend exhibit a smooth behaviour resulting to a Hurst exponent close to unity. Their sum corresponds to the macro-scale time-series, see Fig. 4. In the same Figure, we also depict the mid-scale time-series which is outcome of the summation of the IMFs that exhibit a dual behaviour in their Hurst analysis, i.e., IMFs 4 to 11 for the case of global seismicity and Japan. Here, we have shown that this decomposition is possible both for the global as well as the regional case of Japan. An additional regional example concerns California and the Hurst analysis of the IMFs and the trend is shown in Fig. 10(a). An inspection of this figure shows that the macro-scale time-series comprises of the IMFs 11 to 14 plus the trend, while the micro-scale is the sum of IMFs 1 to 3.

For both global and regional seismicity the constituent time-series of micro-, mid- and macro-scale share similar multifractal characteristics, see Figs 5 and 6. In general, the micro-scale time-series points to antipersistence and exhibits a left-skewed or close to symmetric multifractal spectrum. On the contrary, the mid-scale time-series is persistent with a right-skewed multifractal spectrum and a Fourier spectrum (see Fig. 7) mainly in the range from a few tens to a few hundreds of events. If one takes into account the monthly rate of EQs in the examined catalogs (see Methods), it is easily found that this corresponds to time periods similar to the SES lead time. The latter is another time scale, which can be determined experimentally when measurements of the electric field of the Earth are made as in Greece^48,53^ and Japan^36,54–57^, and is definitely related to the preparation time of strong EQs. The average lead time of SES has already been used^18,20,23,42–44^ as a characteristic scale for the study of seismicity in natural time, giving rise to variability minima of the order parameter of seismicity precursory to strong EQs. Here, we examined whether the mid-scale time-series can be used for the same purpose and a comparison of Figs 8 and 9 shows that indeed similar results can be obtained. At this point, we have to comment on the fact that after 2004, although the alarms shown in these Figures have a strong overlap, this does not hold for the earlier period (cf. the concentration of the alarms after 2004 which leads to the aforementioned strong overlap can be attributed to huge changes of seismic activity, because since 1 January 2004, we have a significantly larger number of EQs, i.e., six, with magnitudes M ≥ 8.4 than before, i.e., one). In order to clarify this point, we plot in Fig. S10 the variabilities *β*_100_, *β*_160_, ${\beta }_{100}^{mid}$, and ${\beta }_{160}^{mid}$ together with the corresponding two alarm time-series. Although these two alarms time-series may have a small overlap, the variabilities exhibit almost simultaneous local minima. Within this framework, it is evident that the mid-scale behaviour of seismicity plays an important role in the minimization of the order parameter fluctuations of seismicity before strong EQs. This also holds for the regional behaviour of seismicity as can be seen in Fig. 10(b–d) which depict the results obtained for California (see Methods). There it has been found^42,43^ that the deepest variability *β*_300_ minimum is identified before the occurrence of the 1992 Landers EQ. Figure 10(b) shows that the same holds (see the blue arrow in Fig. 10) when studying, instead of the whole magnitude time-series, the mid-scale time-series for California. The corresponding ${\beta }_{300}^{mid}$ exhibits its deepest minimum before Landers EQ and almost simultaneous with the one observed upon using *β*_300_.

## Conclusion

All the above, point to the conclusion that the EMD of magnitude time-series uncovers the existence of micro-, mid- and macro-scale component time-series in both global and regional seismicity. Out of these three time-series, here we show that the most useful one for earthquake prediction purposes is the mid-scale time-series. This corresponds to a scale that comprises a number of seismic events that on average occur within a period of around a few months or so. The latter time period compares favourably with the SES lead time, thus strengthening the SES potential to achieve EQ prediction.

## Methods

### The Data Analyzed

The global EQ magnitude time-series comes from the Global Centroid Moment Tensor (CMT) Project^61,62^ that covers global seismicity since 1 January 1976. For EQs that took place before 2011, the 1976 to 2010 CMT catalog was used, whereas for EQs since 1 January 2011 to 1 October 2014 the monthly CMT catalogs have been employed (all these catalogs are publicly available from http://www.globalcmt.org/CMTfiles.html). In accordance with previous studies^13,23^, we considered all EQs of magnitude greater than or equal to *M* = 5.0 (=*M*_*thres*_). This resulted in 38,006 EQs during the concerned period of 38 years and 9 months (1 January 1976 to 1 October 2014). Hence, we have a monthly rate of approximately 80 EQs/month. We note that all EQs with *M* ≥ 8.4 except one (i.e., the 2001 Peru *M* = 8.4 EQ) occurred after 1 January 2004 during a period in which the magnitude completeness threshold is^62^ *M*_*c*_ = 5.0. Concerning the previous period (1976–2003), *M*_*c*_ lies between 5.2 and 5.3 as shown in Fig. 5 of ref.^62^. Repeating the calculation by considering all EQs of magnitude greater than or equal to *M*_*thres*_ = 5.2 or 5.3, we obtained the results shown in Figs S11 and S12, respectively. The results previously obtained for *M*_*thres*_ = 5.0 are not seriously affected as far as the prediction of *M* ≥ 8.4 EQs is concerned. In particular, apart from the minima which are precursory to EQs with *M* ≥ 8.4, we now obtain 11 or 14 minima precursory to smaller EQs (see Tables S1 and S2 for *M*_*thres*_ = 5.2 and 5.3, respectively) compared to 11 for *M*_*thres*_ = 5.0. As for the corresponding *τ* values they are now 25% for *M*_*thres*_ = 5.2 and 32% for *M*_*thres*_ = 5.3 compared to 21% for *M*_*thres*_ = 5.0.

For the regional study of Japan, the Japan Meteorological Agency (JMA) seismic catalogue has been employed as in refs^20,24,38^. We considered all the EQs with magnitude *M*_*JMA*_ ≥ 3.5 in the period from 1983 until the Tohoku EQ occurrence on 11 March 2011, within the area 25°–46°N, 125°–148°E which result to 49,145 events and thus to a monthly rate of approximately 150 EQs/month.

For the regional study of California, the United States Geological Survey Northern California Seismic Network catalog of the Northern California Earthquake Data Center, hereafter called NCEDC, that has been used in refs^42,43^ has been also utilized here. We considered all EQs with *M* ≥ 2.5 reported by NCEDC within the area ${{\rm{N}}}_{31.7}^{45.7}{{\rm{W}}}_{127.5}^{112.1}$ during the 25 year period from 1 January 1979 to 1 January 2004. This leads to 31,832 earthquakes giving rise to an average monthly rate of 106 EQs/month.

### Natural time analysis of seismic catalogs

In a time-series comprising *N* EQ events, the natural time of the *k*-th event of energy *Q*_*k*_ is defined^35^ by *χ*_*k*_ = *k*/*N*. We then study the evolution of the pair (*χ*_*k*_, *p*_*k*_), where ${p}_{k}={Q}_{k}/{\sum }_{n=1}^{N}{Q}_{n}$ is the normalized energy (cf. the energy of each EQ was obtained from the relation^69^ *Q* ∝ 10^1.5*M*^; for Japan *M* was estimated^70^ from the EQ magnitude *M*_*JMA*_ reported by JMA). The approach of a dynamical system to a critical point can be identified^37,38^ by means of the variance *κ*_1_ of natural time *χ* weighted for *p*_*k*_, namely ${\kappa }_{1}={\sum }_{k=1}^{N}{p}_{k}{\chi }_{k}^{2}-{({\sum }_{k=1}^{N}{p}_{k}{\chi }_{k})}^{2}\equiv \langle {\chi }^{2}\rangle -{\langle \chi \rangle }^{2}$. It has been argued^41^ (see also pp. 249–253 of ref.^37^) that the quantity *κ*_1_ can serve as an order parameter of seismicity. To compute the fluctuations of *κ*_1_ we apply the following procedure^18,37^: First, take an excerpt comprising *W* successive EQs from the seismic catalog. We call this excerpt *W*. Second, since at least 6 EQs are needed for calculating reliable *κ*_1_^41^, we form a window of length 6 (consisting of the 1st to the 6th EQ in the excerpt *W*) and compute *κ*_1_ for this window. We perform the same calculation by successively sliding this window through the whole excerpt *W*. Then, we iterate the same process for windows with length 7, 8 and so on up to *W*. We then estimate the average value *μ*(*κ*_1_) and the standard deviation *σ*(*κ*_1_) of the ensemble of the *κ*_1_ values thus obtained. The quantity *β*_*W*_ ≡ *σ*(*κ*_1_)/*μ*(*κ*_1_) is defined^60^ as the variability of *κ*_1_ for this excerpt of length *W* and is assigned to the (*W* + 1) EQ in the catalog, the target EQ. The time evolution of the *β* value can then be pursued by sliding the excerpt *W* through the EQ catalog as shown in Figs 8, 9 and 10.

#### Statistical Significance of precursory *β*_*W*_ minima

The statistical significance of an EQ prediction method based on the precursory *β*_*W*_ minima has been already discussed in refs^23,71^ for the study of the global seismicity by CMT and the regional study of Japan, respectively. For the latter case, it has been shown^71^ by various methods, including that of the receiver operating characteristics, that the probability to obtain such a result by chance is of the order of 10^−5^. As concerns the results depicted in Fig. 8 for the global seismicity, the corresponding probability value to achieve them by chance is 1.4 × 10^−4^ as reported in the Appendix of ref.^23^. These probabilities refer to the prediction problem in both the magnitude and the occurrence time domain.

Taking the view that EQ catalogs are marked point-processes^72,73^ in which the EQ occurrence times are marked by the EQ magnitudes, one might also ask the statistical significance of the EQ prediction method based on the precursory *β*_*W*_ minima for the CMT presented in Fig. 8 if we simply randomly shuffle the marks, i.e., the EQ magnitudes, but keep the EQ occurrence times unchanged. This has been done and three examples of the resulting *β*_*W*_ time-series are shown in Figs S13, S14, and S15. Independently of whether we attempt to find the actually observed occurrence times of the *M* ≥ 8.4 EQs or the EQ occurrence times when a *M* ≥ 8.4 mark appears in the synthetic catalog, an average of 36.8 false alarm minima with unbiased standard deviation of 10.675 has been found in addition to the “precursory” *β*_*W*_ minima. Assuming a Gaussian distribution for the number of the false alarm minima (which is compatible with the Kolmogorov-Smirnov test done), the probability to find only 10 or 11 false alarm minima as found in the Discussion section is below 1% (6 or 8 × 10^−3^, respectively). As mentioned in the previous paragraph, in NTA of seismicity only the order of occurrence of the EQs is preserved giving rise to a time-series in which the inter-occurrence times are not involved.

### EMD

Huang’s Empirical Mode Decomposition^29^, otherwise called the sifting process, is used to extract the low and high frequency components of a signal, i.e., its IMF. The process comprises the following steps^30–33^:

- We create one upper and one lower envelope by connecting the maxima and minima, respectively, of the original signal *X*(*t*) with a cubic spline.
- The mean value *m* of the envelopes is subtracted from the original signal, thus we derive a component *h*(*t*) = *X*(*t*) − *m*.
- Using *h* as our new data, we repeat the process deriving a new component *h*′ etc. When one of these components satisfies the IMF’s definition^29^, a fact that means: 1) the average value of the upper and lower envelope of step a) for each point is zero, 2) the number of extrema and the number of zero crossings must be equal or differ at most by one, the process terminates producing the first IMF. We save the IMF’s data in a new variable *x*_1_ = *h*.
- The IMF extracted above is then subtracted from *X*(*t*). The residual is treated as a new signal which starts the sifting process from the top, thus extracting the second IMF *x*_2_ etc.

When the final residual is a monotonic function, the sifting process terminates and we have derived a set of IMFs and the trend of the original signal. By adding them, we can reconstruct the original signal as $X(t)={\sum }_{i=1}^{n}{x}_{i}+trend$, where *x*_*i*_ denotes the *i*-th IMF.

### EEMD

EEMD introduced by Wu and Huang^63^ is a noise assisted data analysis method in which we decompose the original data *X*(*t*) by repeating steps a)-d) of EMD multiple times for the time-series *X*(*t*) + *w*(*t*). Each time, we add a different white noise time-series *w*(*t*) to the original signal. The EEMD IMF is the mean of the corresponding IMF extracted after every trial. The white noise time-series ideally cancel each other out when computing the mean, thus the final IMF computed will have no added noise (for the computational complexity of EMD and EEMD, see ref.^74^).

### R/S or Hurst analysis

For this analysis, introduced by Hurst^58^, we first construct the profile of the time-series *X*_*k*_ defined by ${y}_{i}={\sum }_{k=1}^{i}({X}_{k}-\bar{X})$, where $\bar{X}$ is the average value of *X*_*k*_ and examine the average value of the ratio of the range *R* over the standard deviation *S* of *y*_*i*_ for various scales *l*. If the data are long-range correlated, the expected value of the ratio *R*/*S* on all partial time series of length *l* scales as $\langle R/S\rangle \propto {l}^{H}$ and *H* is the Hurst exponent. The Hurst exponent *H* is greater than 0.5 for persistent time-series while it is smaller than 0.5 for antipersistent time-series.

### MFDFA

MFDFA introduced by Kantelhardt *et al*.^34^ is a generalization of the Detrended Fluctuation Analysis (DFA)^66,67,75^ for the study of multifractal behaviour in presence of trends^76–78^. The aforementioned profile time-series *y*_*i*_ is divided to *N*_*l*_ non-overlapping segments of equal length *l*, *N*_*l*_ = *N*/*l*. For each segment, a local polynomial trend is determined by least-square fitting (for the present study, a second order polynomial has been used as in ref.^27^) and subtracted from *y*_*i*_ so as to obtain ${\tilde{y}}_{i}$ and therefrom ${F}^{2}(l,v)=\frac{1}{l}\,{\sum }_{i=1}^{l}{\tilde{y}}_{i}^{2}$. Averaging over all segments results in the *q*-th order fluctuation function ${F}_{q}(l)={\{\frac{1}{{N}_{l}}{\sum }_{v=1}^{{N}_{l}}{F}^{2}{(l,v)}^{q/2}\}}^{1/q}$ where the index variable *q* can be assigned with any real value except zero (for *q* = 2, DFA is retrieved). The scaling behaviour of time-series is determined by analyzing the log-log plots of *F*_*q*_(*l*) versus *l* for each value of *q* in order to determine a set of generalized Hurst exponents *h*(*q*) according to the relation *F*_*q*_(*l*) ∝ *l*^*h*(*q*)^. From *h*(*q*), we can estimate^34^ the singularity spectrum^79^ *f*(*a*) from the relations: *τ*(*q*) = *qh*(*q*) − 1, $a=a(q)\equiv \frac{\partial \tau (q)}{\partial q}$ and *f*(*a*) = *aq* − *τ*(*q*).

## Electronic supplementary material

Supplementary Information
