# Supplementary material for: Micro-scale, mid-scale, and macro-scale in global seismicity identified by empirical mode decomposition and their multifractal characteristics
Source: Sci Rep. 2018 Jun 15;8:9206. doi: 10.1038/s41598-018-27567-y (PMC6003985; doi:10.1038/s41598-018-27567-y)
Supplement: Supplementary file 1 — Supplementary Information [file 41598_2018_27567_MOESM1_ESM.pdf]

# Supplementary Information for the paper entitled: Micro-scale, mid-scale, and macro-scale in global seismicity identified by empirical mode decomposition and their multifractal characteristics

**Nicholas V. Sarlis**[1,2,*], **Efthimios S. Skordas**[1,2], **Apostolis Mintzelas**[1], **and Konstantina A. Papadopoulou**[1]

[1]Section of Solid State Physics, Department of Physics, National and Kapodistrian University of Athens, Panepistimiopolis, Zografos 157 84, Athens, Greece
[2]Solid Earth Physics Institute, Department of Physics, National and Kapodistrian University of Athens, Panepistimiopolis, Zografos 157 84, Athens, Greece
[*]nsarlis@phys.uoa.gr

## ABSTRACT

Supplementary Figures S1 to S15 as well as Supplementary Tables S1 and S2 are provided.

## Supplementary Figures and Tables

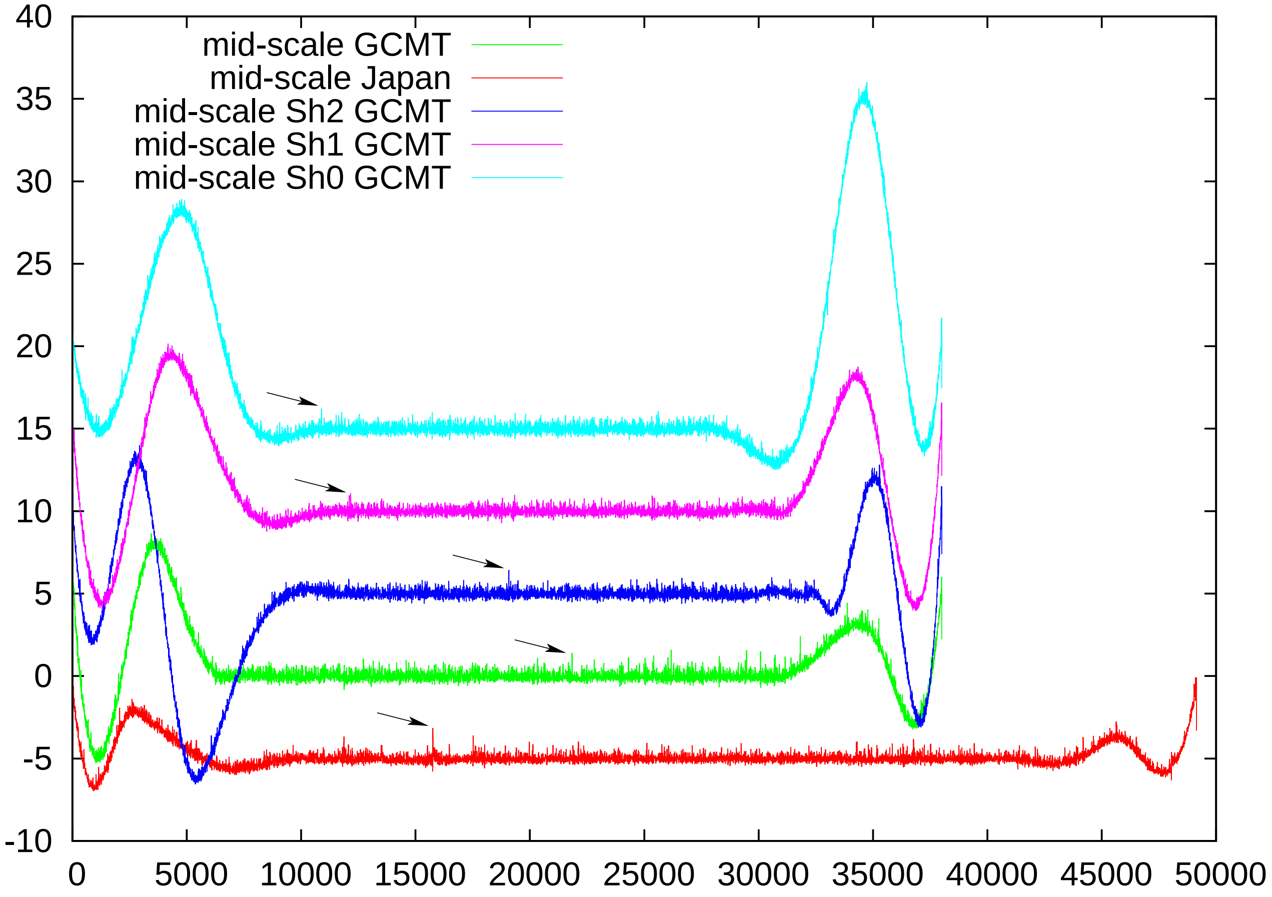


**Figure S1. Comparison of mid-scale time-series.** The mid-scale time-series for global seismicity (green), Japan (red) as well as the mid-scale time-series obtained from the EMD of time-series in which the EQ magnitudes of the global seismicity are randomly shuffled (the same time-series are used later in Figs.S13, S14, and S14) displaced vertically by constant amounts. Not only the modes of the edge effects are different but also some extreme fluctuations indicated by arrows occur at different points.

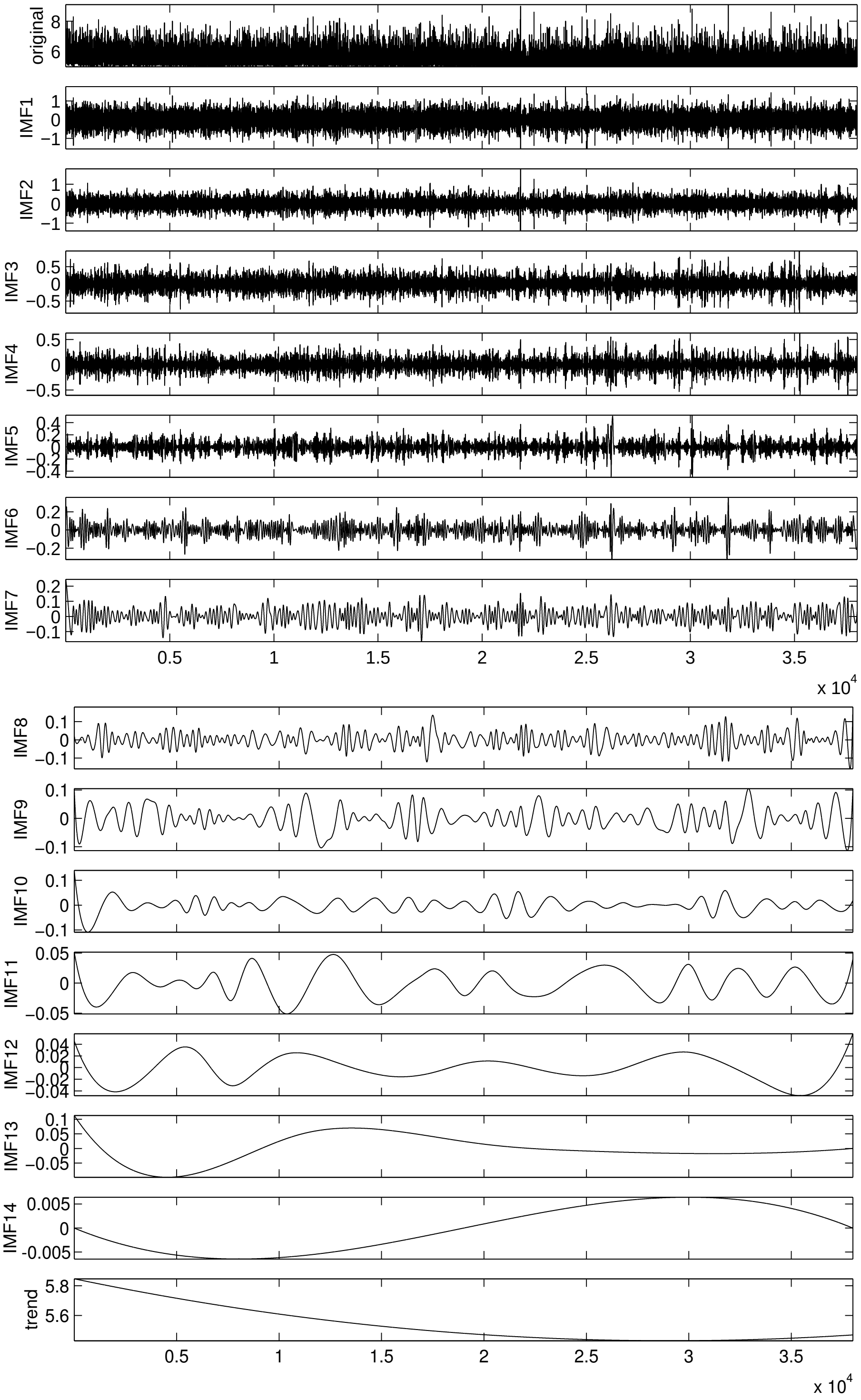


**Figure S2. Fast EMD of the magnitude time-series of global seismicity in 14 IMFs and a trend.** Here we decompose the global seismicity time-series in 14 IMFs and a trend after following Ref.[63] of the main text and employing the Matlab code package freely available from `http://rcada.ncu.edu.tw` and especially the matlab code `FEEMD.zip` discussed in detail in Ref.[74] of the main text.

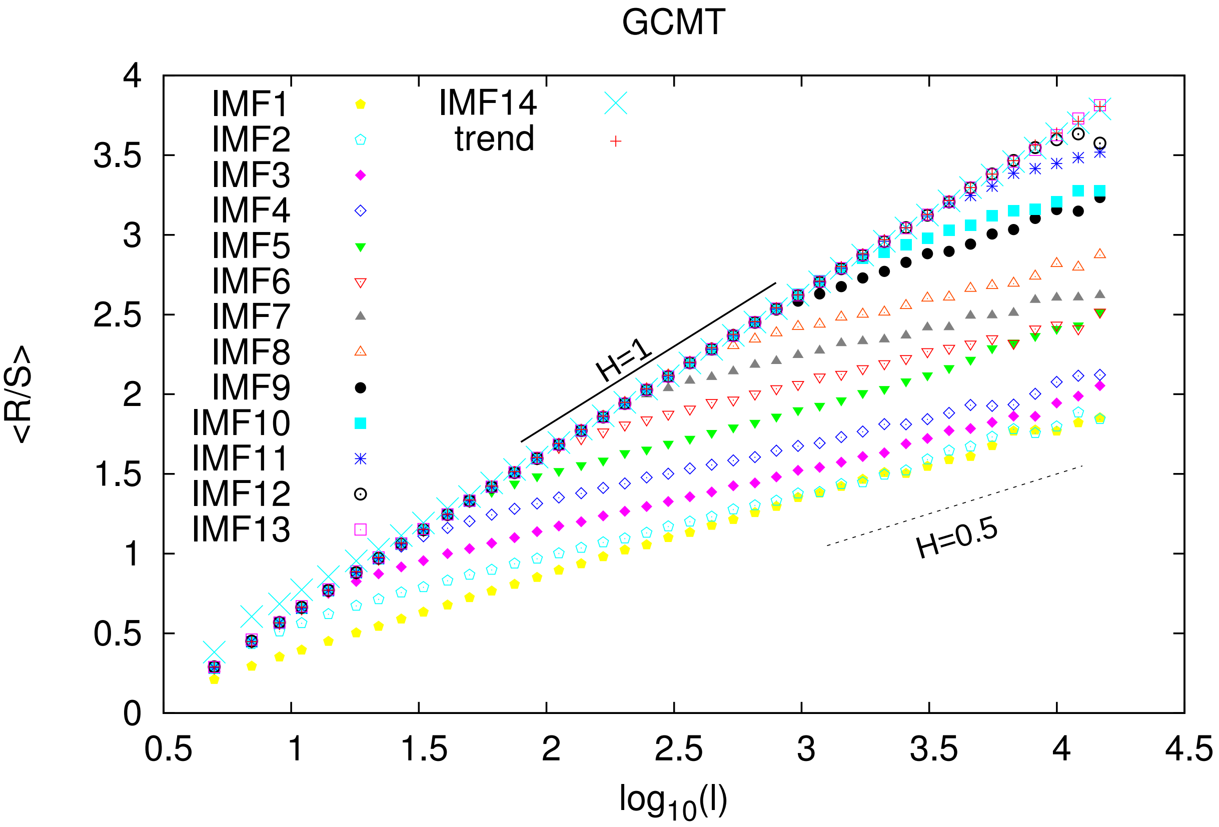


**Figure S3. Hurst analysis for the IMFs and the trend depicted in Figure S2.** The solid and the dashed line correspond to $H = 1$ and $H = 0.5$, respectively. Here, the IMFs 13 and 14 as well as the trend exhibit a straight line behaviour with unit slope, while IMFs 3 to 12 show a cross-over in which the slope changes from $H = 1$ to values that may be even smaller than $H = 0.5$.

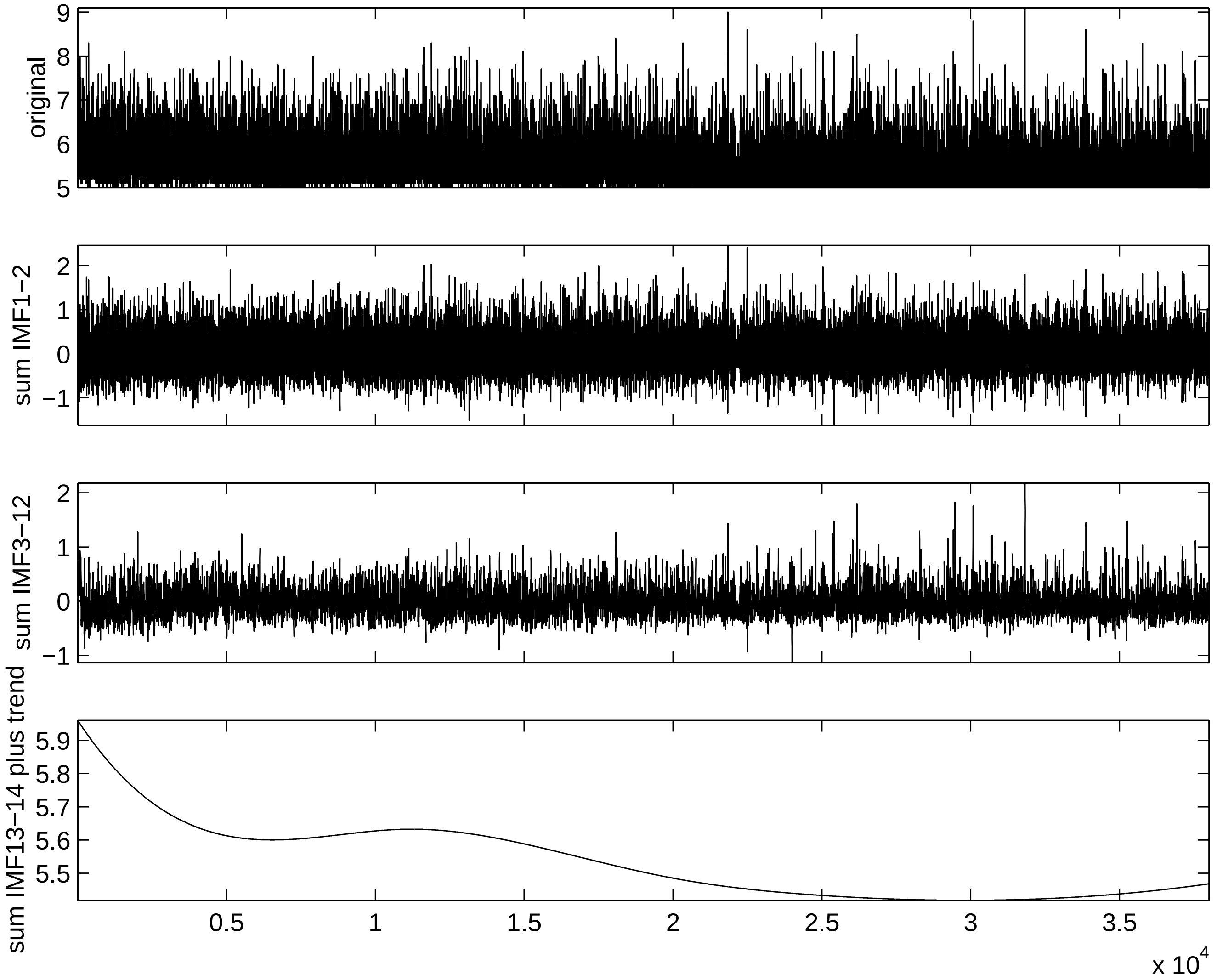


**Figure S4. Separation of magnitude time-series into three components when following Figure S2.** Here, we decompose the GCMT magnitude time-series according to the results of Figure S3 into a micro-scale (composed of IMFs 1 and 2), a mid-scale (composed of the sum of IMFs 3 to 12) and a macro-scale time-series (composed of the sum of IMFs 13, 14 and the trend).

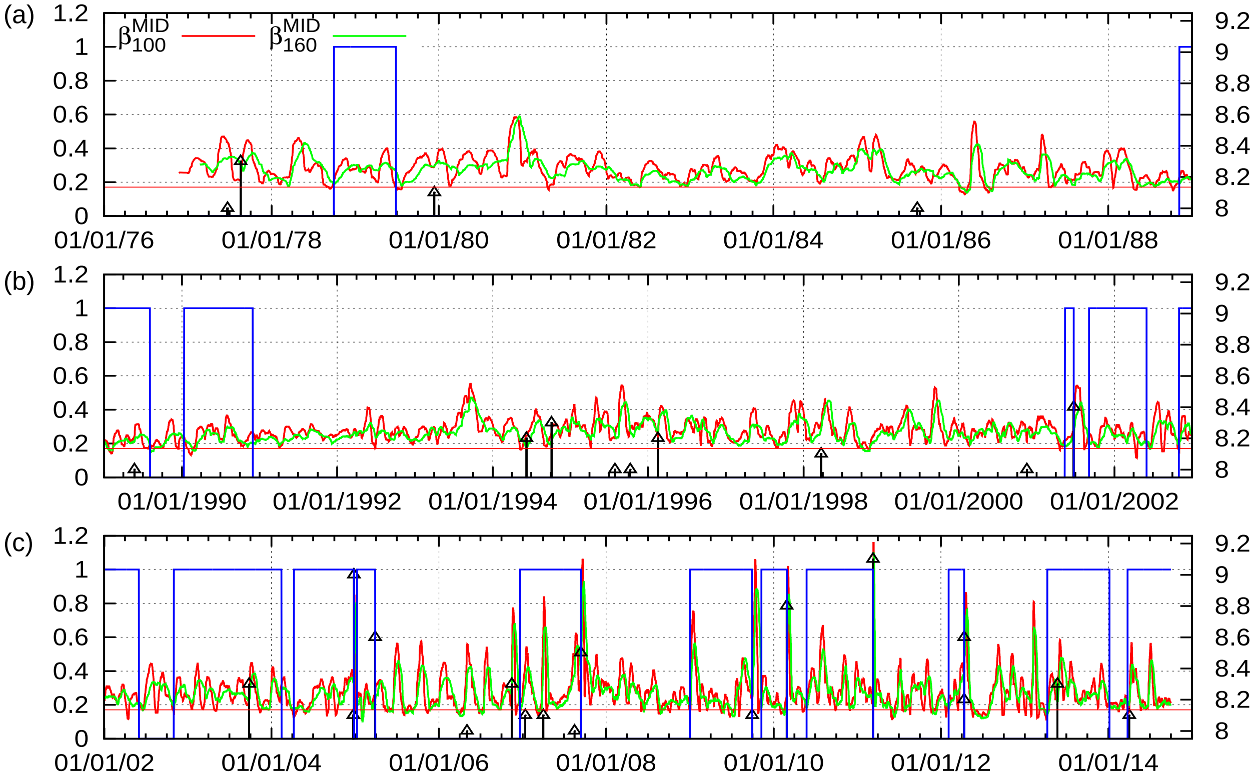


**Figure S5. Analysis of the mid-scale time-series (MID: sum of IMFs 3 to 12 of Figure S4) of the global seismicity in natural time.** The variabilities (left scale) $\beta_{100}^{MID}$ (red) and $\beta_{160}^{MID}$ (green) versus conventional time for the periods: (a) 1 January 1976 to 31 December 1988, (b) 1 January 1989 to 31 December 2002, and (c) 1 January 2002 to 1 October 2014. The thin blue line corresponds to the alarm (1=on and 0=off, left scale) lasting nine months after the occurrence of $\min(\beta_{160}^{MID})$ when using $(\beta_0, r_1, r_2) = (0.171, 1.15, 1.37)$ for the prediction of the occurrence times of EQs with $M \geq 8.4$ which are shown with the vertical lines ending at black triangles (right scale). The percentange of the total alarm time is $\tau = 25\%$. The horizontal red line corresponds to $\beta_0 = 0.171$.

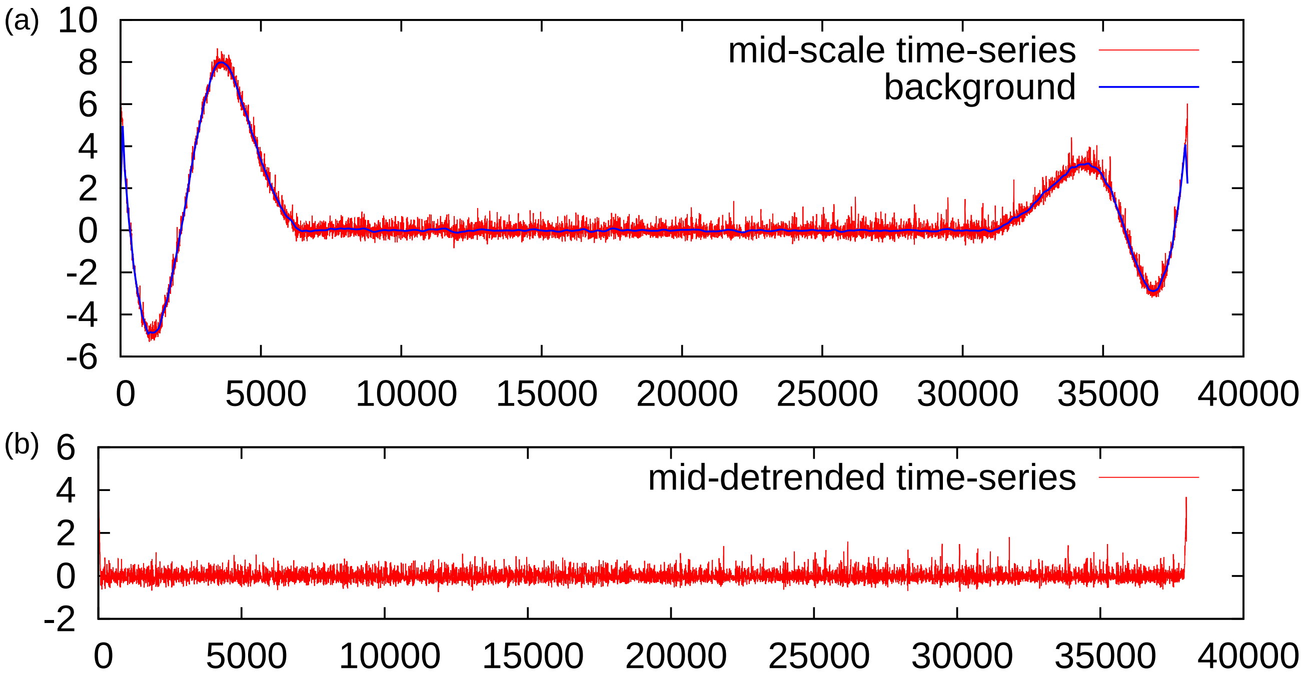


**Figure S6. Detrending of the mid-scale time-series of global seismicity of Figure 4 of the main text.** Panel (a) depicts the mid-scale time-series (red) of global seismicity (see Figure 4 of the main text) together with a backgound (blue) determined by Bezier smoothing when using the standard computer code `GNUPLOT`, while panel (b) shows the residual, labeled mid-detrended, time-series. It is obvious that the strong edge effects have been removed and only the first and the last 100 points are affected.

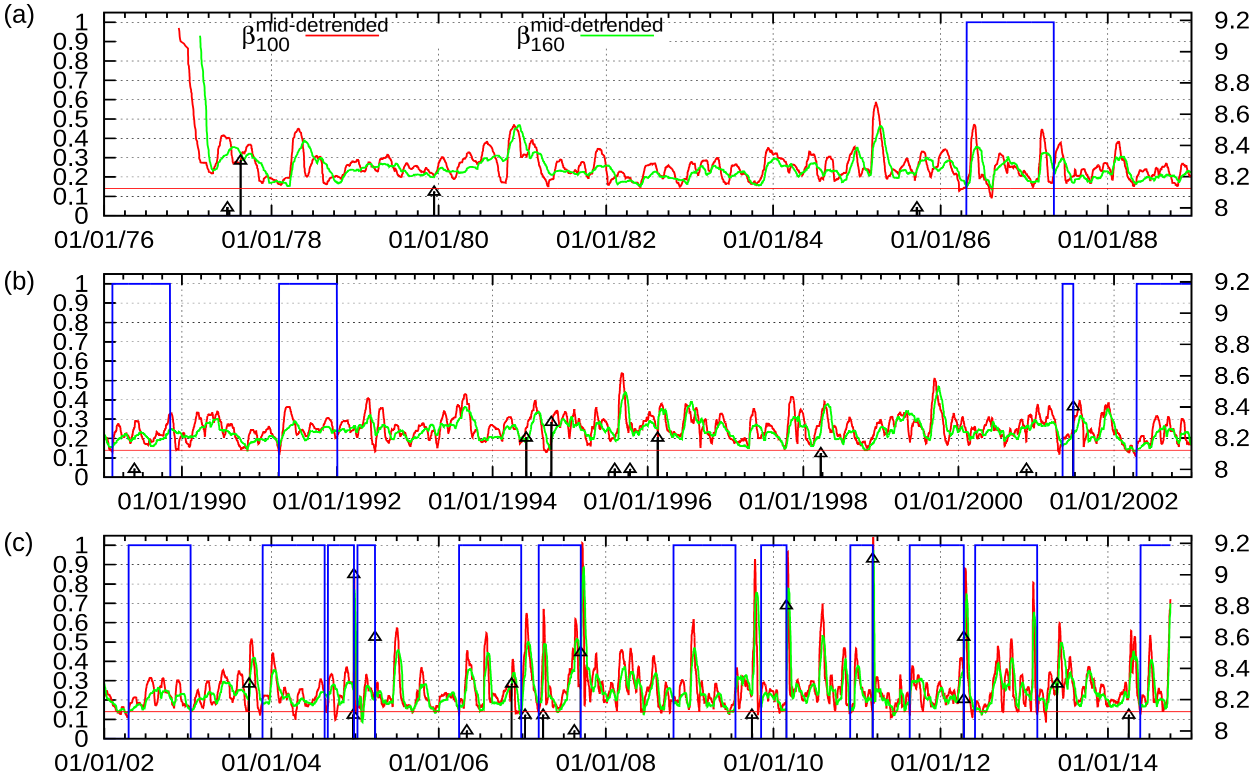


**Figure S7.** **Analysis of the detrended mid-scale time-series of the global seismicity (see Figure S6) in natural time.** The variabilities (left scale) $\beta_{100}^{mid-detrended}$ (red) and $\beta_{160}^{mid-detrended}$ (green) versus conventional time for the periods: (a) 1 January 1976 to 31 December 1988, (b) 1 January 1989 to 31 December 2002, and (c) 1 January 2002 to 1 October 2014. The thin blue line corresponds to the alarm (1=on and 0=off, left scale) lasting nine months after the occurrence of $\min(\beta_{160}^{mid-detrended})$ when using $(\beta_0, r_1, r_2) = (0.140, 1.13, 1.54)$ for the prediction of the occurrence times of EQs with $M \geq 8.4$ which are shown with the vertical lines ending at black triangles (right scale). The percentange of the total alarm time is $\tau = 24\%$. The horizontal red line corresponds to $\beta_0 = 0.140$.

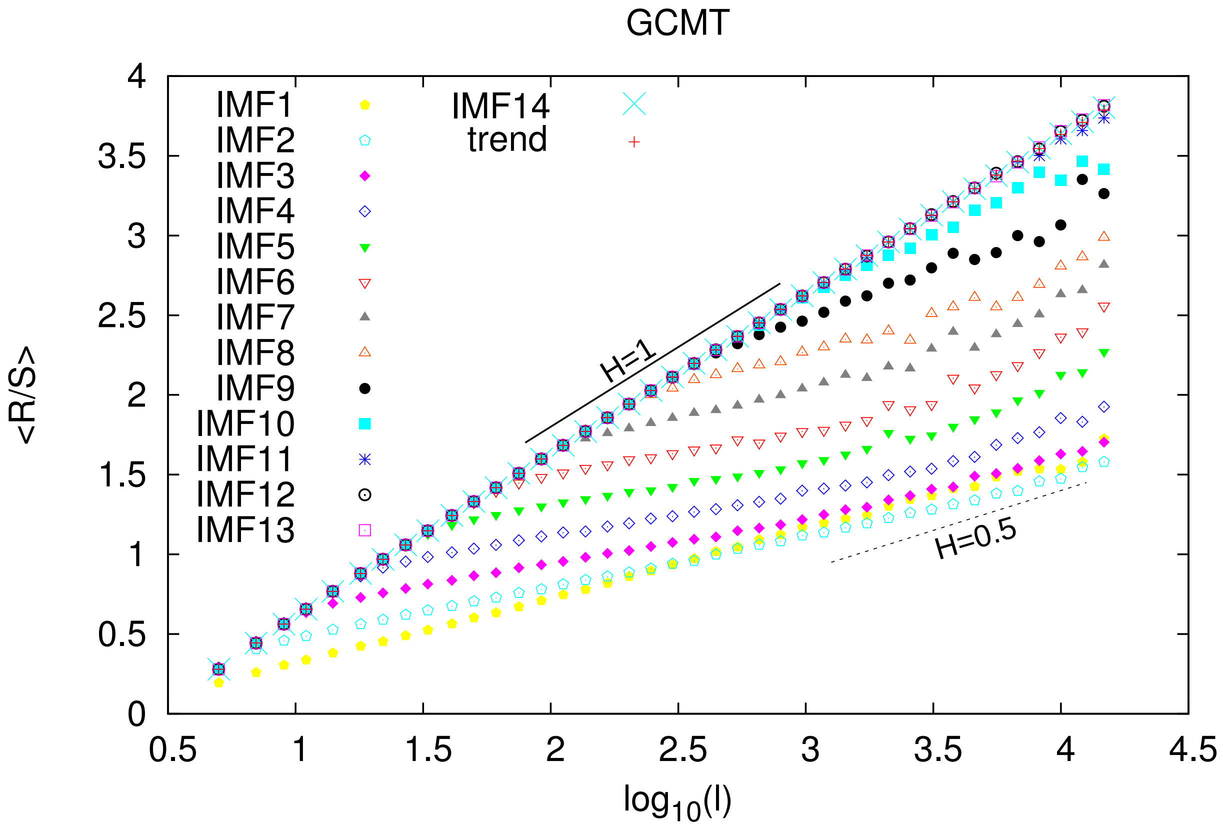


**Figure S8.** **Hurst analysis for the IMFs and the trend determined by EEMD for the global seismicity.** The solid and the dashed line correspond to $H = 1$ and $H = 0.5$, respectively. As in Figure 3 of the main text, the IMFs 12, 13 and 14 as well as the trend exhibit a straight line behaviour with unit slope, while IMFs 4 to 11 show a cross-over. In accordance to Ref.[63] of the main text for EEMD the following procedure has been employed $10^2$ times: to the original global seismicity time-series, white Gaussian noise time-series of amplitude 0.2 standard deviations of the original data have been added and the resulting time-series has been decomposed by EMD. For the cases EMD resulted in 14 IMFs and a trend, the corresponding mean time-series have been determined and analyzed here.

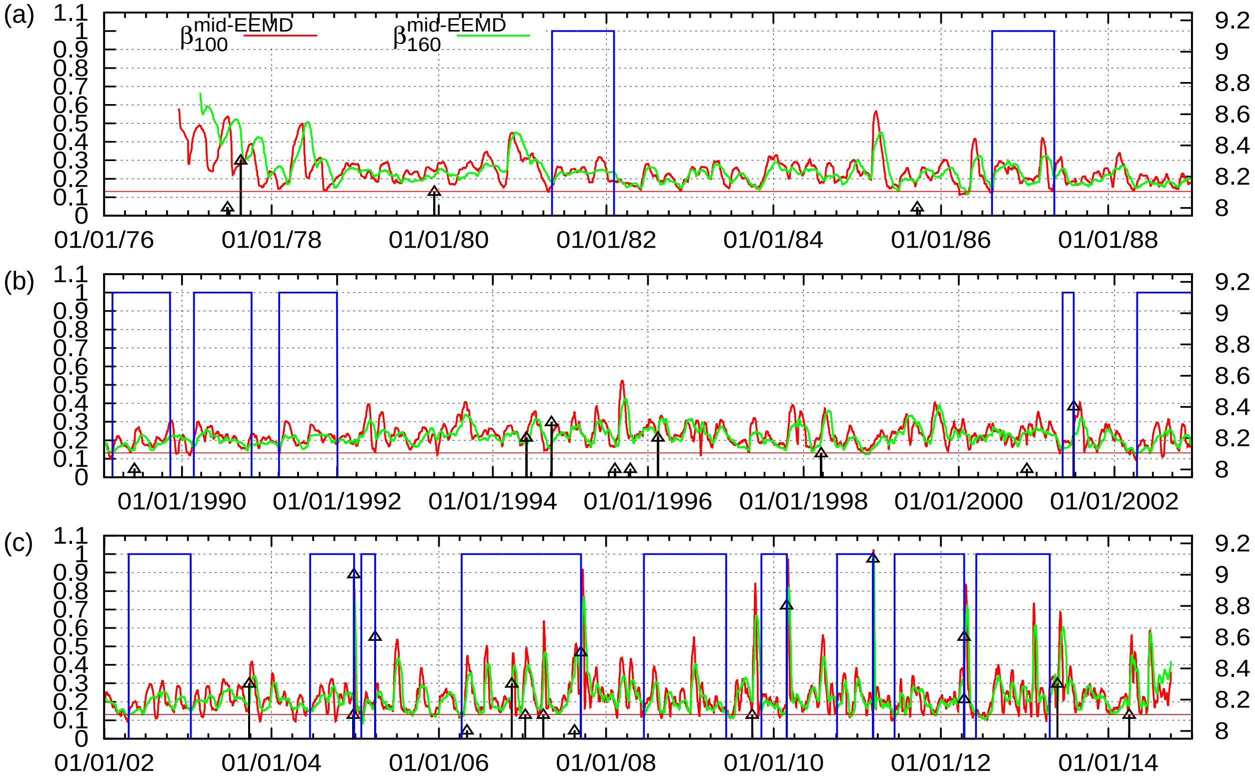


**Figure S9. Analysis of the mid-scale time-series of the global seismicity in natural time as determined by EEMD (see Figure S8).** The variabilities (left scale) $\beta_{100}^{mid-EEMD}$ (red) and $\beta_{160}^{mid-EEMD}$ (green) versus conventional time for the periods: (a) 1 January 1976 to 31 December 1988, (b) 1 January 1989 to 31 December 2002, and (c) 1 January 2002 to 1 October 2014. The thin blue line corresponds to the alarm (1=on and 0=off, left scale) lasting nine months after the occurrence of $\min(\beta_{160}^{mid-EEMD})$ when using $(\beta_0, r_1, r_2) = (0.132, 1.03, 1.44)$ for the prediction of the occurrence times of EQs with $M \geq 8.4$ which are shown with the vertical lines ending at black triangles (right scale). The percentange of the total alarm time is $\tau = 27\%$. The horizontal red line corresponds to $\beta_0 = 0.132$.

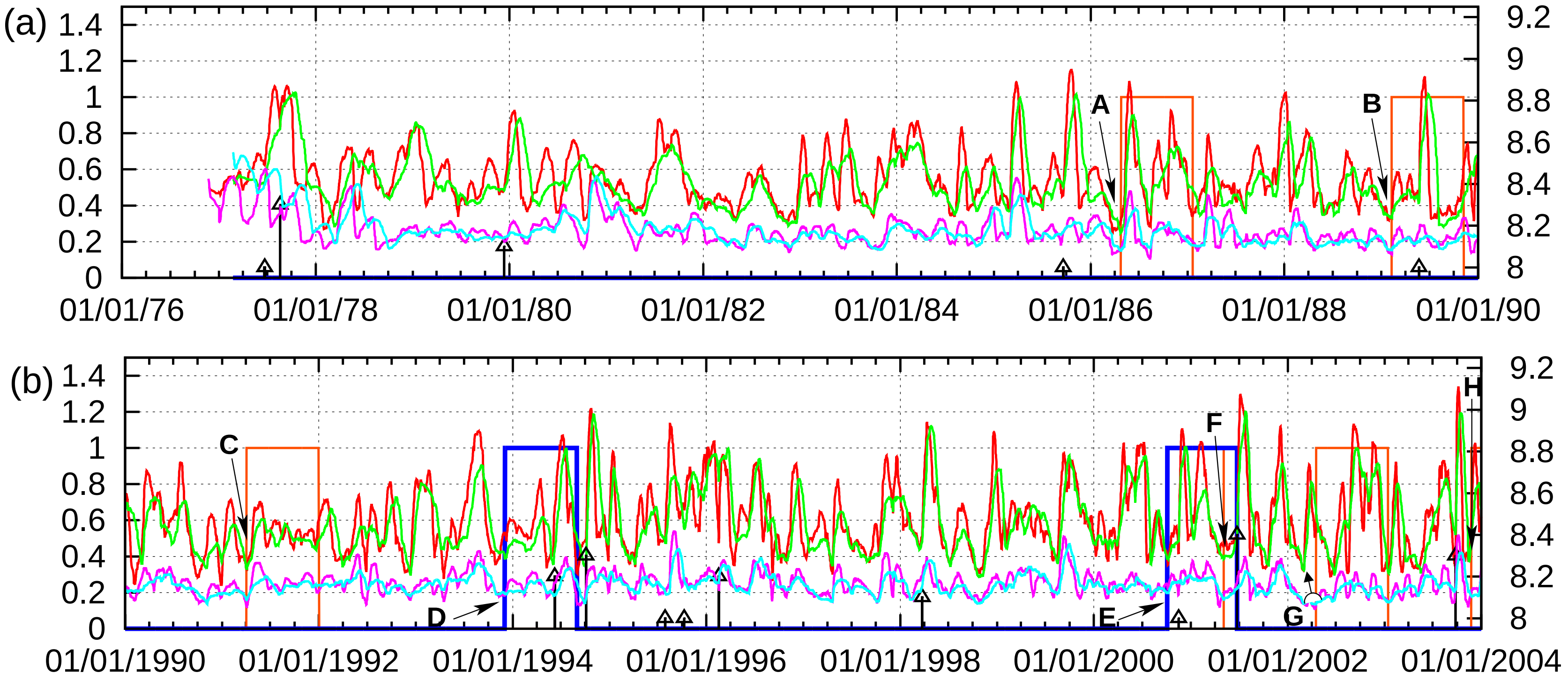


**Figure S10. Comparison of the alarms shown in Figs.8 and 9 of the main text.** The variabilities (left scale) $\beta_{100}$ (red), $\beta_{160}$ (green), $\beta_{100}^{mid}$ (magenta) and $\beta_{160}^{mid}$ (cyan) versus conventional time for the periods: (a) 1 January 1976 to 31 December 1989, (b) 1 January 1990 to 31 December 2003. The thick blue line corresponds to the alarm (1=on and 0=off, left scale) lasting nine months after the occurrence of $\min(\beta_{160})$ when using $(\beta_0, r_1, r_2) = (0.353, 1.060, 1.135)$ for the prediction of the occurrence times of EQs with $M \geq 8.4$ which are shown with the vertical lines ending at black triangles (right scale), while the thinner orange line corresponds to the alarm lasting nine months after the occurrence of $\min(\beta_{160}^{mid})$ when using $(\beta_0, r_1, r_2) = (0.140, 1.13, 1.54)$ for the prediction of the occurrence times of EQs with $M \geq 8.4$. Although these two alarms may have a small overlap, the arrows (A, B, C, to H) indicate that the variabilities shown exhibit almost simultaneous local minima. The selection of local minima as precursory, and hence the initiation of an alarm, depends on the statistics of $\beta_{100}$, $\beta_{160}$ and $\beta_{100}^{mid}$, $\beta_{160}^{mid}$ which is different for the original and the mid-scale time-series. This difference is also reflected in the different values of $(\beta_0, r_1, r_2)$ used in each case. Here we focus on the period before 2004, since after 2004 there is significant overlap of the alarms.

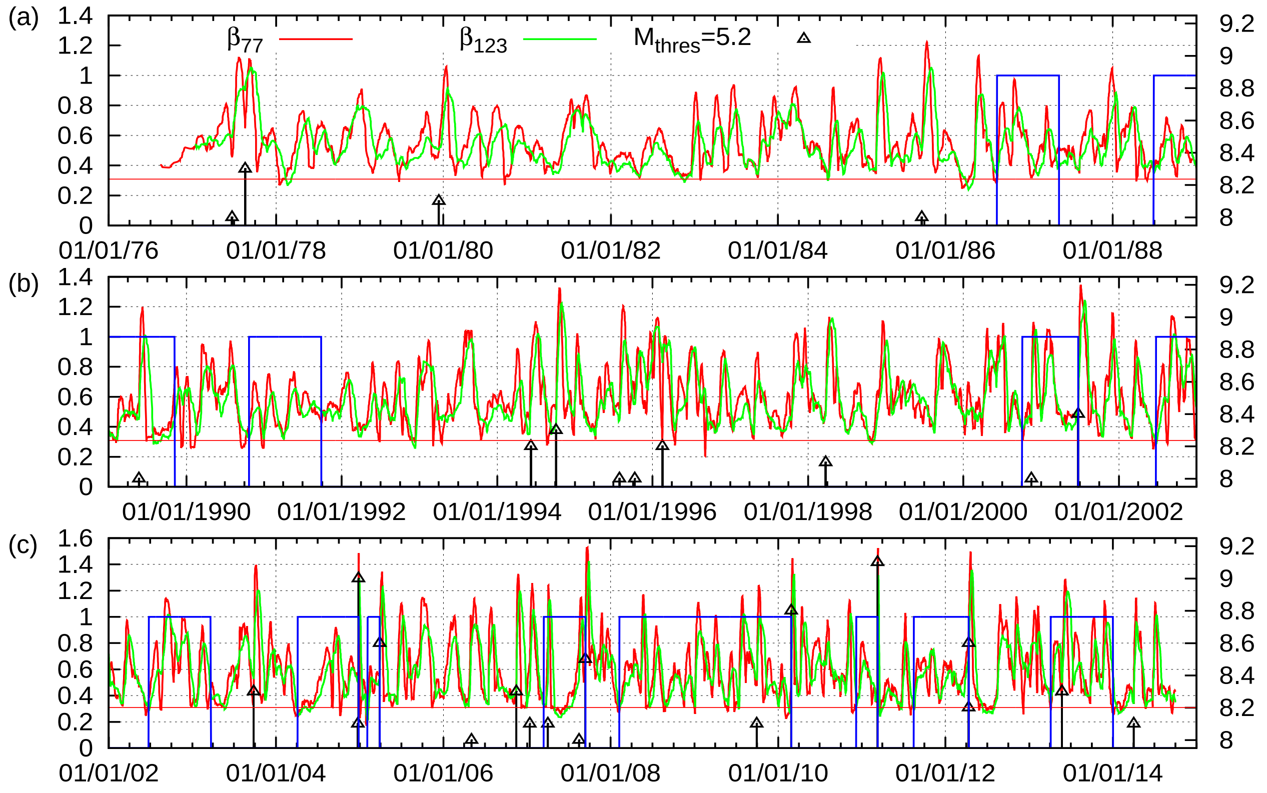


**Figure S11. Natural time analysis of global seismicity for $M_{thres} = 5.2$.** In this case, the total number of EQs is 29,274 ( i.e., 77% of the original 38,006 EQs for $M_{thres} = 5.0$) and hence the $W$ values corresponding to the characteristic time scale of a few months become $W = 77$ and $W = 123$ (instead of $W$ =100 and $W$ =160, respectively). The variabilities (left scale) $\beta_{77}$ (red) and $\beta_{123}$ (green) versus conventional time for the periods: (a) 1 January 1976 to 31 December 1988, (b) 1 January 1989 to 31 December 2002, and (c) 1 January 2002 to 1 October 2014. The thin blue line corresponds to the alarm (1=on and 0=off, left scale) lasting nine months after the occurrence of $\min(\beta_{123})$ when using $(\beta_0, r_1, r_2) = (0.309, 1.05, 1.25)$ for the prediction of the occurrence times of EQs with $M \geq 8.4$ which are shown with the vertical lines ending at black triangles (right scale). The percentange of the total alarm time is $\tau = 25\%$. The horizontal red line corresponds to $\beta_0 = 0.309$.

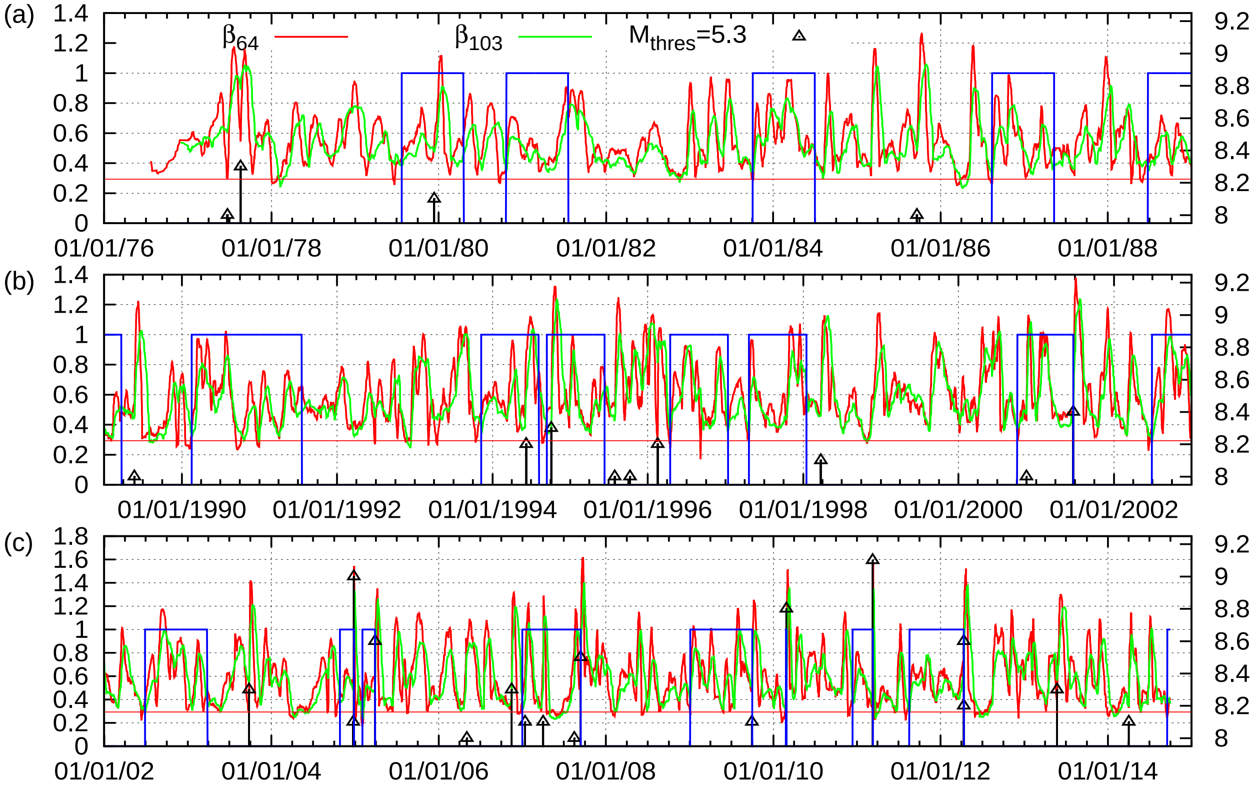


**Figure S12. Natural time analysis of global seismicity for $M_{thres} = 5.3$.** In this case, the total number of EQs is 24,380 ( i.e., 64% of the original 38,006 EQs for $M_{thres} = 5.0$) and hence the $W$ values corresponding to the characteristic time scale of a few months become $W = 64$ and $W = 103$ (instead of $W$ =100 and $W$ =160, respectively). The variabilities (left scale) $\beta_{64}$ (red) and $\beta_{103}$ (green) versus conventional time for the periods: (a) 1 January 1976 to 31 December 1988, (b) 1 January 1989 to 31 December 2002, and (c) 1 January 2002 to 1 October 2014. The thin blue line corresponds to the alarm (1=on and 0=off, left scale) lasting nine months after the occurrence of $\min(\beta_{103})$ when using $(\beta_0, r_1, r_2) = (0.293, 1.15, 1.51)$ for the prediction of the occurrence times of EQs with $M \geq 8.4$ which are shown with the vertical lines ending at black triangles (right scale). The percentange of the total alarm time is $\tau = 32\%$. The horizontal red line corresponds to $\beta_0 = 0.293$.

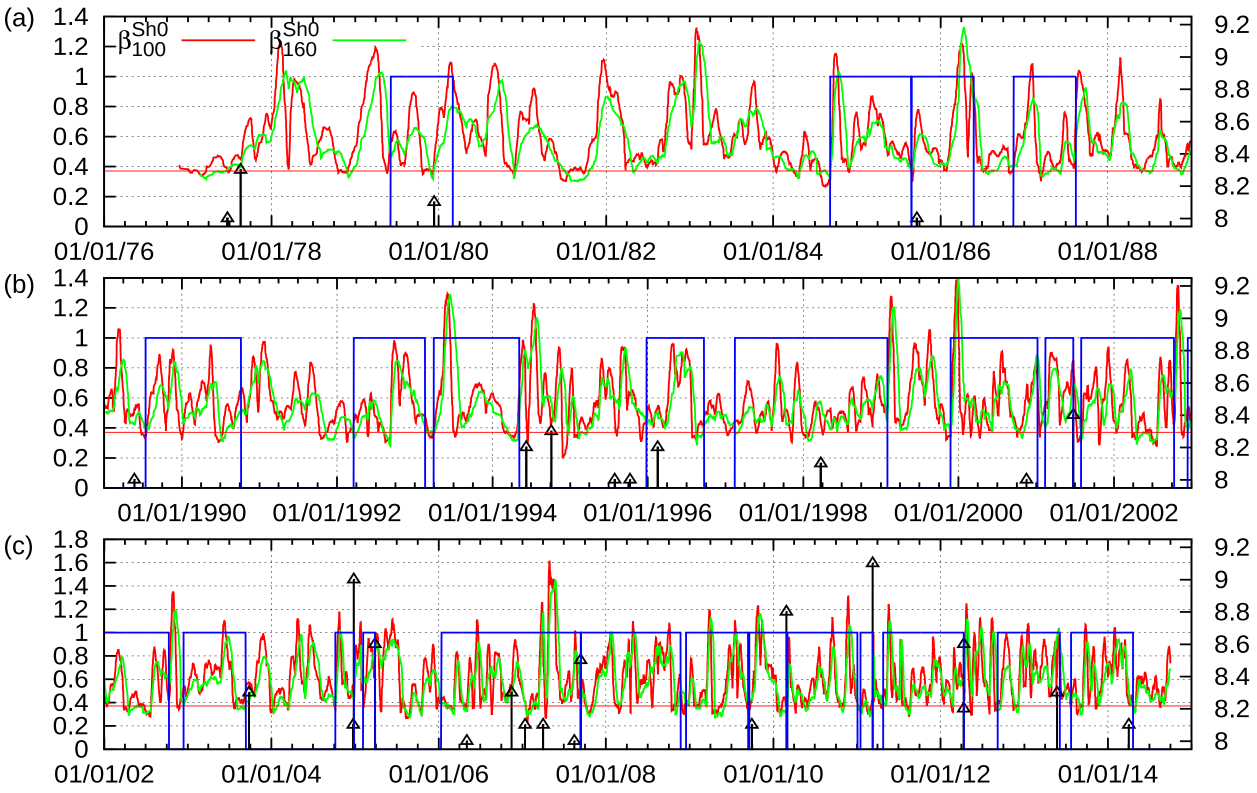


**Figure S13. Natural time analysis of synthetic catalogs where the correlations between magnitudes have been destroyed.** Here the variabilities have been determined by using a randomly shuffled copy (labeled Sh0) of the EQ magnitudes reported in CMT while keeping the EQ occurrence times unchanged. These variabilities (left scale) $\beta_{100}$ (red) and $\beta_{160}$ (green) are plotted versus conventional time for the periods: (a) 1 January 1976 to 31 December 1988, (b) 1 January 1989 to 31 December 2002, and (c) 1 January 2002 to 1 October 2014. The thin blue line corresponds to the alarm (1=on and 0=off, left scale) lasting nine months after the occurrence of $\min(\beta_{160}^{Sh0})$ when using $(\beta_0, r_1, r_2) = (0.371, 1.03, 1.51)$ for the prediction of the actually observed (e.g., see Tables S1 and S2) occurrence times of EQs with $M \geq 8.4$ which are shown with the vertical lines ending at black triangles (right scale). The percentange of the total alarm time is $\tau = 54\%$.

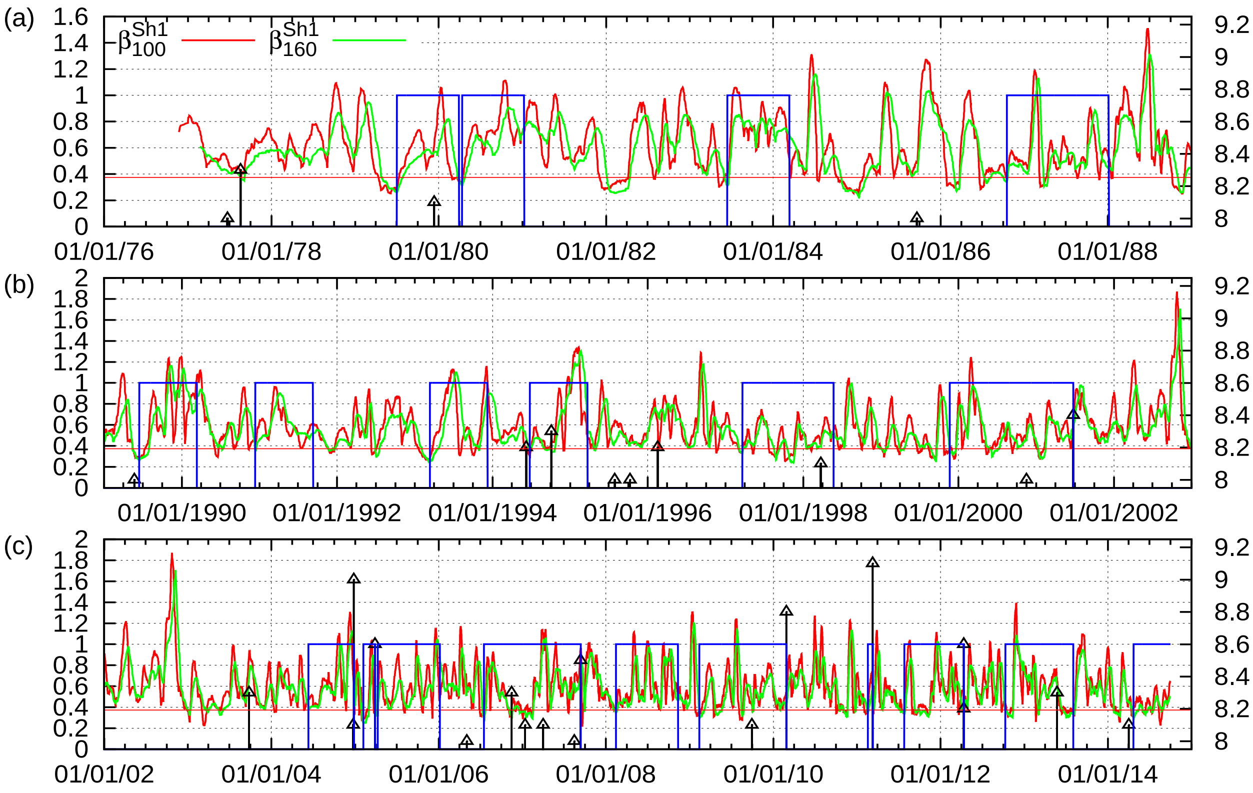


**Figure S14. Natural time analysis of synthetic catalogs where the correlations between magnitudes have been destroyed.** Here the variabilities have been determined by using another randomly shuffled copy (labeled Sh1) of the EQ magnitudes reported in CMT while keeping the EQ occurrence times unchanged. The variabilities (left scale) $\beta_{100}$ (red) and $\beta_{160}$ (green) are plotted versus conventional time for the periods: (a) 1 January 1976 to 31 December 1988, (b) 1 January 1989 to 31 December 2002, and (c) 1 January 2002 to 1 October 2014. The thin blue line corresponds to the alarm (1=on and 0=off, left scale) lasting nine months after the occurrence of $\min(\beta^{Sh1}_{160})$ when using $(\beta_0, r_1, r_2) = (0.374, 0.93, 1.04)$ for the prediction of the actually observed (e.g., see Tables S1 and S2) occurrence times of EQs with $M \geq 8.4$ which are shown with the vertical lines ending at black triangles (right scale). The percentange of the total alarm time is $\tau = 41\%$.

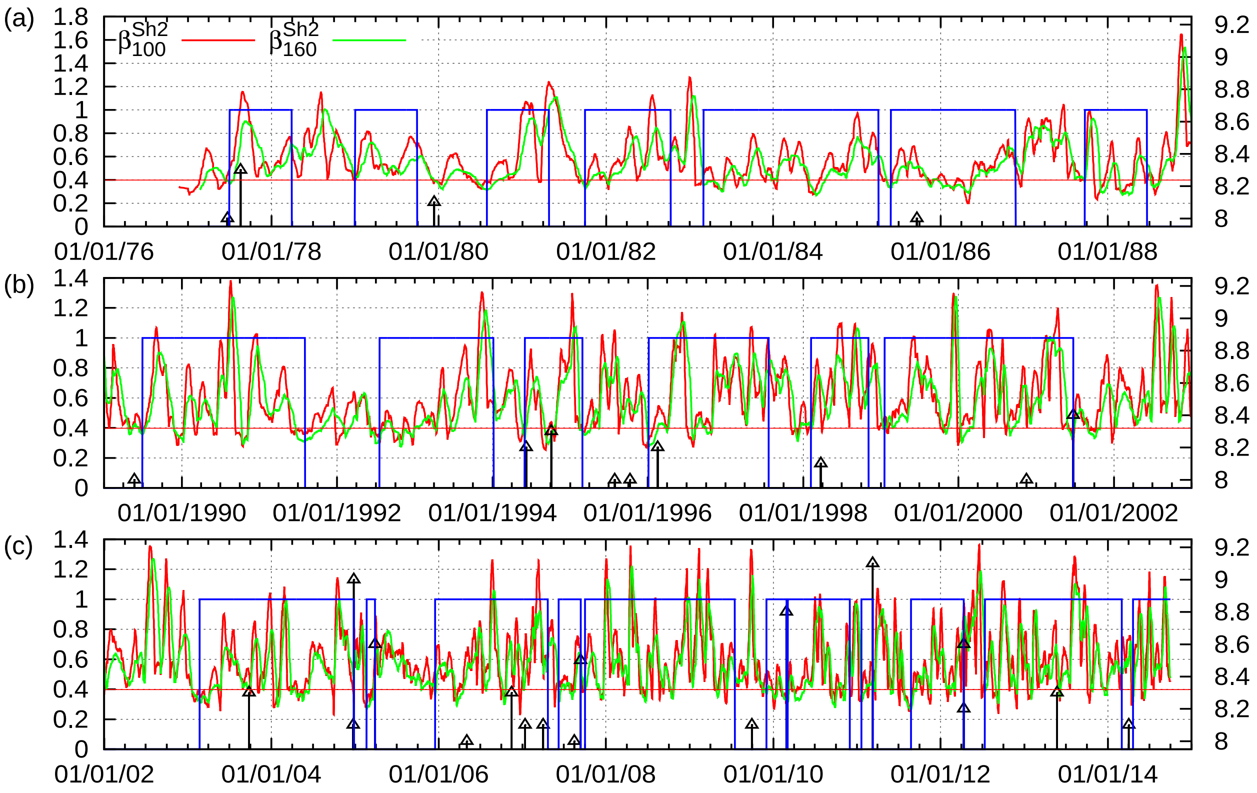


**Figure S15. Natural time analysis of synthetic catalogs where the correlations between magnitudes have been destroyed.** Here the variabilities have been determined by using yet another randomly shuffled copy (labeled Sh2) of the EQ magnitudes reported in CMT while keeping the EQ occurrence times unchanged. The variabilities (left scale) $\beta_{100}$ (red) and $\beta_{160}$ (green) are plotted versus conventional time for the periods: (a) 1 January 1976 to 31 December 1988, (b) 1 January 1989 to 31 December 2002, and (c) 1 January 2002 to 1 October 2014. The thin blue line corresponds to the alarm (1=on and 0=off, left scale) lasting nine months after the occurrence of $\min(\beta_{160}^{Sh2})$ when using $(\beta_0, r_1, r_2) = (0.398, 0.96, 1.14)$ for the prediction of the actually observed (e.g., see Tables S1 and S2) occurrence times of EQs with $M \geq 8.4$ which are shown with the vertical lines ending at black triangles (right scale). The percentange of the total alarm time is $\tau = 69\%$.

| EQ date | Lat. ($^o$N) | Long. ($^o$E) | $M$ | min($\beta_{77}$) | min($\beta_{123}$) | $r$ | $\Delta t_{123}$ (months) |
|---|---|---|---|---|---|---|---|
| 19861020 | -28.10 | -176.43 | 7.7 | 0.298(19860801) | 0.334(19860814) | 1.120 | 2.2 |
| ” | ” | ” | 7.7 | 0.285(19860810) | ” | 1.172 | 2.2 |
| 19880810 | -10.21 | 160.77 | 7.5 | 0.299(19880530) | 0.348(19880628) | 1.163 | 1.4 |
| 19890523 | -52.24 | 160.20 | 8.0 | 0.294(19890204) | 0.314(19890210) | 1.070 | 3.4 |
| 19910422 | 9.68 | -83.08 | 7.6 | 0.260(19900920) | 0.320(19901023) | 1.228 | 6.0 |
| ” | ” | ” | 7.6 | 0.260(19901228) | 0.319(19901230) | 1.226 | 3.8 |
| 20010623 | -16.26 | -73.64 | **8.4** | 0.308(20001006) | 0.386(20001004) | 1.250 | 8.7 |
| 20021103 | 63.52 | -147.44 | 7.8 | 0.251(20020613) | 0.284(20020625) | 1.133 | 4.4 |
| 20041226 | 3.30 | 95.78 | **9.0** | 0.236(20040405) | 0.254(20040405) | 1.073 | 8.8 |
| 20050328 | 2.09 | 97.11 | **8.6** | 0.171(20050130) | 0.212(20050204) | 1.237 | 1.7 |
| 20070912 | -4.44 | 101.37 | **8.5** | 0.306(20070317) | 0.325(20070315) | 1.059 | 6.0 |
| 20080512 | 31.00 | 103.32 | 7.9 | 0.275(20080204) | 0.294(20080208) | 1.071 | 3.1 |
| 20090103 | -0.41 | 132.88 | 7.7 | 0.281(20080820) | 0.321(20080903) | 1.145 | 4.1 |
| ” | ” | ” | 7.7 | 0.277(20080825) | ” | 1.158 | 4.1 |
| 20090929 | -15.49 | -172.10 | 8.1 | 0.261(20090103) | 0.311(20090103) | 1.192 | 9.0 |
| 20090929 | -15.49 | -172.10 | 8.1 | 0.283(20090527) | 0.322(20090528) | 1.135 | 4.1 |
| 20100227 | -35.85 | -72.71 | **8.8** | 0.276(20090624) | 0.302(20090712) | 1.094 | 7.7 |
| ” | ” | ” | **8.8** | 0.223(20100204) | 0.264(20100222) | 1.183 | 0.2 |
| 20110311 | 38.32 | 142.37 | **9.1** | 0.271(20101121) | 0.315(20101208) | 1.162 | 3.1 |
| 20120411 | 2.33 | 93.06 | **8.6** | 0.249(20110802) | 0.292(20110816) | 1.170 | 8.0 |
| 20130524 | 54.89 | 153.22 | 8.3 | 0.302(20130405) | 0.353(20130405) | 1.168 | 1.6 |

**Table S1.** The EQs that are preceded within 9 months from the variability minima identified upon employing $M_{thres} = 5.2$ and studying the global seismicity using the parameters $(\beta_0, r_1, r_2) = (0.309, 1.05, 1.25)$ for the prediction of the occurrence times of all EQs with $M \geq 8.4$. The latter are typed in boldface. $\Delta t_{123}$ corresponds to the time period that elapsed from the observation of $\min(\beta_{123})$ and the EQ occurrence measured in months. The dates of EQs as well as the dates of minima appearance are shown in the format YYYYMMDD. When considering that a variability minimum can be identified by its $\min(\beta_{123})$, there exist 11 minima which are precursory to EQs of magnitudes $M$ in the range $7.3 \leq M \leq 8.3$.

| EQ date | Lat. ($^o$N) | Long. ($^o$E) | $M$ | min($\beta_{64}$) | min($\beta_{103}$) | $r$ | $\Delta t_{103}$ (months) |
|---|---|---|---|---|---|---|---|
| 19791212 | 1.60 | -79.36 | 8.1 | 0.255(19790622) | 0.384(19790724) | 1.503 | 4.7 |
| 19810525 | -48.79 | 164.36 | 7.6 | 0.268(19800924) | 0.347(19801022) | 1.298 | 7.2 |
| 19831130 | -6.89 | 72.12 | 7.7 | 0.282(19831004) | 0.338(19831004) | 1.200 | 1.9 |
| 19861020 | -28.10 | -176.43 | 7.7 | 0.260(19860814) | 0.304(19860814) | 1.170 | 2.2 |
| 19880810 | -10.21 | 160.77 | 7.5 | 0.275(19880530) | 0.346(19880625) | 1.259 | 1.5 |
| 19900716 | 15.66 | 121.23 | 7.7 | 0.241(19900208) | 0.319(19900217) | 1.325 | 5.0 |
| 19910422 | 9.68 | -83.08 | 7.6 | 0.233(19900921) | 0.294(19901021) | 1.259 | 6.1 |
| 19940609 | -13.83 | -67.56 | 8.2 | 0.281(19931013) | 0.358(19931109) | 1.274 | 7.1 |
| 19941004 | 43.71 | 147.33 | 8.3 | 0.274(19940822) | 0.335(19940914) | 1.224 | 0.7 |
| 19960610 | 51.56 | -177.63 | 7.9 | 0.242(19960416) | 0.356(19960416) | 1.474 | 1.8 |
| 19971205 | 54.84 | 162.04 | 7.8 | 0.287(19970421) | 0.341(19970421) | 1.186 | 7.6 |
| 20010623 | -16.26 | -73.64 | **8.4** | 0.292(20001005) | 0.371(20001004) | 1.267 | 8.7 |
| 20021103 | 63.52 | -147.44 | 7.8 | 0.227(20020612) | 0.282(20020629) | 1.238 | 4.2 |
| ” | ” | ” | 7.8 | 0.227(20020613) | ” | 1.238 | 4.2 |
| 20041226 | 3.30 | 95.78 | **9.0** | 0.258(20041008) | 0.378(20041027) | 1.463 | 2.0 |
| 20050328 | 2.09 | 97.11 | **8.6** | 0.164(20050201) | 0.236(20050202) | 1.437 | 1.8 |
| 20070912 | -4.44 | 101.37 | **8.5** | 0.278(20061212) | 0.320(20070101) | 1.152 | 8.5 |
| 20090929 | -15.49 | -172.10 | 8.1 | 0.268(20090103) | 0.355(20090103) | 1.324 | 9.0 |
| 20100227 | -35.85 | -72.71 | **8.8** | 0.198(20100207) | 0.299(20100225) | 1.509 | 0.1 |
| 20110311 | 38.32 | 142.37 | **9.1** | 0.262(20101123) | 0.356(20101213) | 1.358 | 2.9 |
| 20120411 | 2.33 | 93.06 | **8.6** | 0.221(20110801) | 0.289(20110819) | 1.307 | 7.9 |
| 20140917 | 13.76 | 144.43 | 6.8 | 0.246(20140915) | 0.361(20140917) | 1.466 | 0.0 |

**Table S2.** The EQs that are preceded within 9 months from the variability minima identified upon employing $M_{thres} = 5.3$ and studying the global seismicity using the parameters $(\beta_0, r_1, r_2) = (0.293, 1.15, 1.51)$ for the prediction of the occurrence times of all EQs with $M \geq 8.4$. The latter are typed in boldface. $\Delta t_{103}$ corresponds to the time period that elapsed from the observation of min$(\beta_{103})$ and the EQ occurrence measured in months. The dates of EQs as well as the dates of minima appearance are shown in the format YYYYMMDD. When considering that a variability minimum can be identified by its min$(\beta_{103})$, there exist 14 minima which are precursory to EQs of magnitudes $M$ in the range $6.8 \leq M \leq 8.3$.
